# Major Radiations in the Evolution of Caviid Rodents: Reconciling Fossils, Ghost Lineages, and Relaxed Molecular Clocks

**DOI:** 10.1371/journal.pone.0048380

**Published:** 2012-10-29

**Authors:** María Encarnación Pérez, Diego Pol

**Affiliations:** CONICET, Museo Paleontológico Egidio Feruglio, Trelew, Chubut Province, Argentina; Fordham University, United States of America

## Abstract

**Background:**

Caviidae is a diverse group of caviomorph rodents that is broadly distributed in South America and is divided into three highly divergent extant lineages: Caviinae (cavies), Dolichotinae (maras), and Hydrochoerinae (capybaras). The fossil record of Caviidae is only abundant and diverse since the late Miocene. Caviids belongs to Cavioidea *sensu stricto* (Cavioidea *s.s*.) that also includes a diverse assemblage of extinct taxa recorded from the late Oligocene to the middle Miocene of South America (“eocardiids”).

**Results:**

A phylogenetic analysis combining morphological and molecular data is presented here, evaluating the time of diversification of selected nodes based on the calibration of phylogenetic trees with fossil taxa and the use of relaxed molecular clocks. This analysis reveals three major phases of diversification in the evolutionary history of Cavioidea *s.s.* The first two phases involve two successive radiations of extinct lineages that occurred during the late Oligocene and the early Miocene. The third phase consists of the diversification of Caviidae. The initial split of caviids is dated as middle Miocene by the fossil record. This date falls within the 95% higher probability distribution estimated by the relaxed Bayesian molecular clock, although the mean age estimate ages are 3.5 to 7 Myr older. The initial split of caviids is followed by an obscure period of poor fossil record (refered here as the Mayoan gap) and then by the appearance of highly differentiated modern lineages of caviids, which evidentially occurred at the late Miocene as indicated by both the fossil record and molecular clock estimates.

**Conclusions:**

The integrated approach used here allowed us identifying the agreements and discrepancies of the fossil record and molecular clock estimates on the timing of the major events in cavioid evolution, revealing evolutionary patterns that would not have been possible to gather using only molecular or paleontological data alone.

## Introduction

Estimating the timing of evolutionary diversification events is the field of major interaction between paleontology and molecular biology. During the last two decades the alternative evolutionary timescales for different taxonomic groups were the focus of intense debates between paleontologists and molecular biologists [1]–[6]. The divergence estimates from molecular data do not always coincide with the first appearances in the fossil record, as molecules often estimates dates too old, and the fossil record underestimates the actual dates. Recent attempts to reconcile the two sources of information [7]–[10] aimed to increase the interaction and reciprocal illumination between these two sources, rather than highlighting the conflict or disagreement between paleontological and molecular clock estimates. The integration of both sources of evidence can reveal patterns on the time and mode of the evolutionary history of a group that would not be evident using only fossils or DNA sequences.

Rodents provide an interesting case for analyzing the interaction between fossils and molecules, as this is a diverse group with a relatively complete fossil record. Rodents are the most diverse group of mammals at present, which include more than 2256 species representing 41% of all mammals [11] and their evolution has been intensively studied in recent years [12]–[20]. South American rodents belong to Caviomorpha [21] and evolved during the last 45 million years from hystricognath forms that invaded South America (most likely from Africa) during the Paleogene [13], [20], [22]–[29]. Caviomorphs underwent an extraordinary evolutionary radiation that made this group the rodent clade with the greatest morphological and ecological disparity, including the broadest range of body size within Rodentia [30]–[34]. Examples of the diversity of caviomorph rodents are porcupines (Erethizontoidea), coypus, degus, and spiny rats (Octodontoidea), viscachas, chinchillas, and pacaranas (Chinchilloidea), and capybaras, maras, cavies (or ‘guinea pigs’), and pacas (Cavioidea) [34], [35].

Within caviomorphs, Cavioidea is crucial for understanding the diversification of South American rodents given that it includes the greatest morphological disparity (e.g., digitigrades [36]–[39]), inhabits a broad range of ecosystems (e.g., semiaquatic, open grasslands, dry steppes, forest edges, wetlands, rocky ledges [40]), displays diverse levels of sociality [41], and includes the largest living rodent [35], [40], [42].

Different authors, however, have variously interpreted the taxonomic content of Cavioidea. The most inclusive and traditional conception of the group includes four families: Dasyproctidae (agouties), Cuniculidae (pacas), Caviidae (cavies and maras), and Hydrochoeridae (capybaras) [34], [35], [43]–[45]. Patterson and Wood [46] recognized Cavioidea *sensu stricto* (Cavioidea *s.s.*) clustering the extant Caviidae and Hydrochoeridae together with a diverse assemblage of primitive taxa of the extinct family Eocardiidae, given the presence of unique dental and mandibular modifications (e.g., heart-shaped occlusal surface, hypsodonty, reduced lateral deflection of the angular process). Recent phylogenetic analyses of this group based on morphological characters [47] corroborated the monophyly of Cavioidea *s.s.* but retrieved a paraphyletic arrangement of “eocardiids” as successive sister taxa of the crown-group comprised of cavies, maras, and capybaras.

Regarding the relationships of the extant lineages, the availability of DNA sequences of extant species of Cavioidea *s.s.* has provided a wealth of new data to understand more thoroughly the relationships of the group, such as the close affinities of the capybara (*Hydrochoerus*) and cavies (i.e., *Cavia*) that have been corroborated in broad-scale phylogenetic analyses of hystricognath rodents [13], [18], [20]. The most detailed molecular phylogenetic analysis of cavioid rodents [41] retrieved a deeply nested position of *Hydrochoerus* within the multiple representatives of cavies and maras used in that study, being the sister group of the Rock cavy, *Kerodon*. This result was incongruent with traditional morphological classification schemes, but has been subsequently corroborated by phylogenetic studies based on morphological characters [47]–[49].

Therefore the clade Caviidae can be applied to the cluster of the crown-group of three major living lineages: cavies (Caviinae), maras (Dolichotinae), and capybaras (Hydrochoerinae). These three major lineages of extant caviids are well differentiated from a morphological and ecological perspective. Cavies are usually small-bodied taxa that inhabit a variety of environments (e.g., open grassland, forest edge, swamps) and they feed on diverse types of plants. Maras, instead, are much larger, adapted to cursorial habits with elongated hind limbs, and exclusively inhabit arid areas with coarse grass or scattered shrubs. Capybaras are not only the largest rodents alive but are also characterized by their highly apomorphic dentition (e.g., multilaminated molariforms with extremely deep flexus/ids) and inhabits densely vegetated areas around freshwater bodies [50]. Here we follow the taxonomic proposal of recognizing Caviidae as the crown-group formed by these three distinct living lineages [34], [51]. Cavioidea *sensu stricto* represents the clade formed by Caviidae and its stem group (the more basal and paraphyletic “eocardiids”), and Cavioidea as an even more inclusive group that also includes Cuniculidae and Dasyproctidae, the two other lineages leading to the extant pacas and agouties.

The major focus of this contribution is the analysis of the timing and diversification patterns in the evolutionary history of Cavioidea *s.s.* and its crown-group Caviidae, using the information of the fossil record and molecular clock estimates (evaluating the impact of different calibration constraints based on a critical use of the available fossil record). The fossil record of Cavioidea *s.s.* includes a large diversity of extinct species recorded in South America since the late Oligocene (24.5–29 Ma). The fossil evidence indicates that Cavioidea *s.s.* diversified after the arrival of rodents in South America [52], [53], formed an important component of the vertebrate fauna, and occupied a broad range of ecological niches during the rest of the Cenozoic Era. Traditionally, the early evolution of the group was interpreted as a case of gradual transformation [52], [54], [55] starting from the low-crowned and mesodont primitive forms recorded in the Oligocene and ending with the diversification of high-crowned euhypsodont forms that appear in the fossil record at the early Miocene (15.7–16.5 Ma). Fossil members of the crown-group Caviidae, however, appear later in the fossil record, by the late middle Miocene (ca. 11.8–13.5 Ma) and became morphologically and taxonomically diverse since the late Miocene (ca. 6.1–9.07 Ma). The recent morphological phylogenetic studies of these extinct and extant forms [47]–[49] have implications for understanding the tempo and mode of the evolution of Caviodea *s.s.*–previous hypotheses on the group, including the traditional interpretation of gradual evolution, need to be revisited within an explicit phylogenetic context.

Furthermore, the availability of molecular data for species of Caviidae also allowed exploring the divergence time of this clade using different approaches to the molecular clock [13], [20], [56]–[59], some of which proposed the divergence time of Caviidae occurred up to 25 Ma, several million years before the first appearance of the group in the fossil record. The availability of molecular and morphological phylogenies with extensive taxon sampling of fossil taxa, and the extensive fossil record of Cavioidea *s.s.* provide an interesting test-case to evaluate the congruence between divergence estimates based on the fossil record and the molecular clock.

Here we present new phylogenetic results based on a morphological dataset that expands previously published evidence [47]–[49] and a molecular dataset of four genes. The results of a simultaneous parsimony analysis of morphological and molecular data and a Bayesian analysis of the molecular partition are used to evaluate the diversification patterns in the evolutionary history of Cavioidea *s.s.* Finally, we compare the timing of these events as inferred from the fossil record evidence and through the use of relaxed molecular clocks [60], assessing the possible causes of conflict on divergence times inferred from the available paleontological evidence as well as from the molecular clock estimates. This offers a more complete understanding of the evolutionary tempo and mode of Cavioidea *s.s.*

## Results

**Figure 1:**
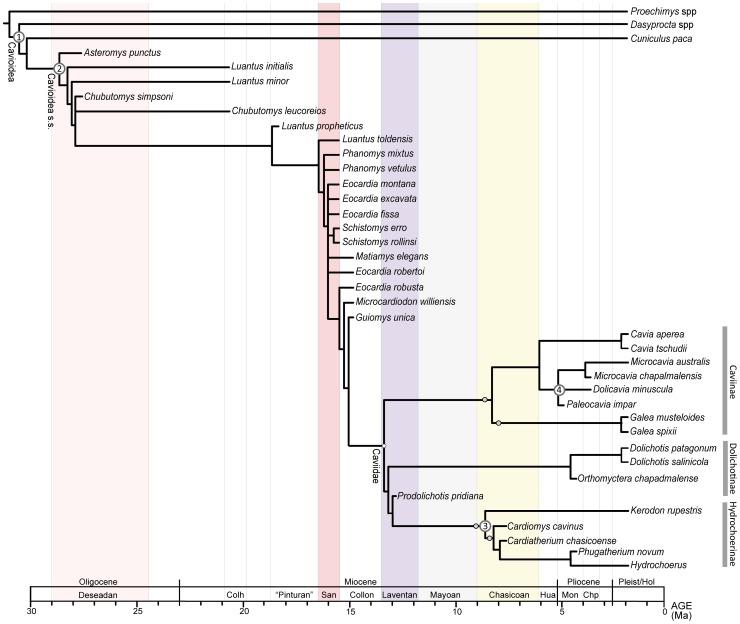
Reduced strict consensus of the parsimony combined phylogenetic analysis of morphological and molecular dataset using TNT. Small yellow circles show the multiple alternative positions in the most parsimonious trees of the late Miocene caviid *Allocavia* (pruned from the reduced consensus tree). Numbered nodes on the tree indicate the calibration constraints used in the molecular clock estimates (see Calibrated Nodes). The phylogenetic tree is calibrated against geological time based on the first occurrence of fossil taxa in the fossil record. The most relevant South American Land Mammal Ages are highlighted in color.

**Figure 2:**
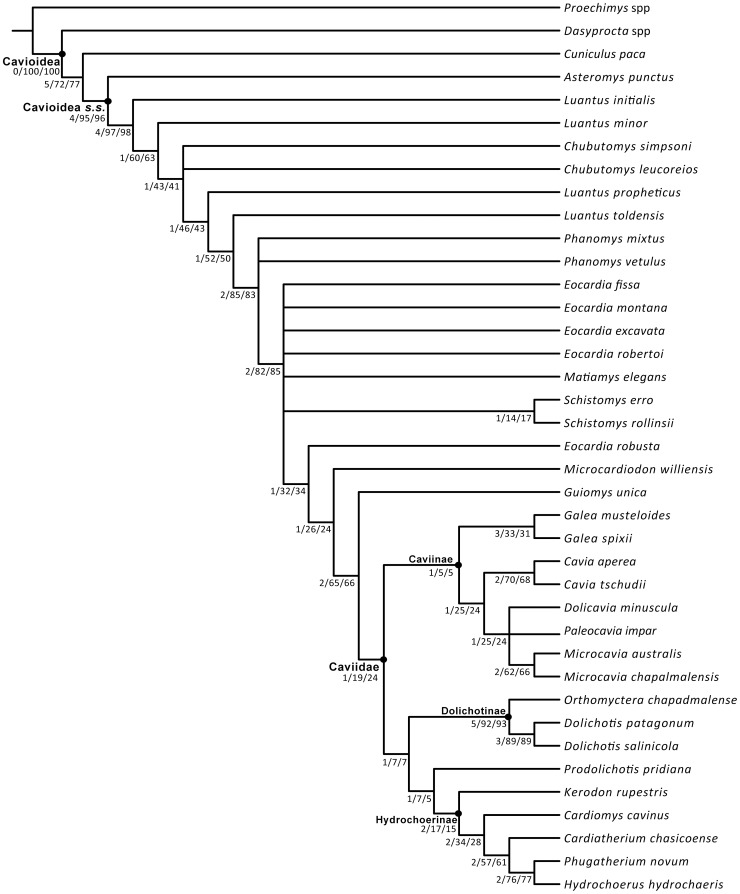
Nodal support values of the parsimony combined phylogenetic analysis of morphological and molecular dataset using TNT. Three values are indicated for each node, the first of them is the Bremer support value, the second is the absolute frequency of each node in the bootstrap replicates and the third is the absolute frequency in the jackknife replicates (see Document S1 for further details).

**Figure 3:**
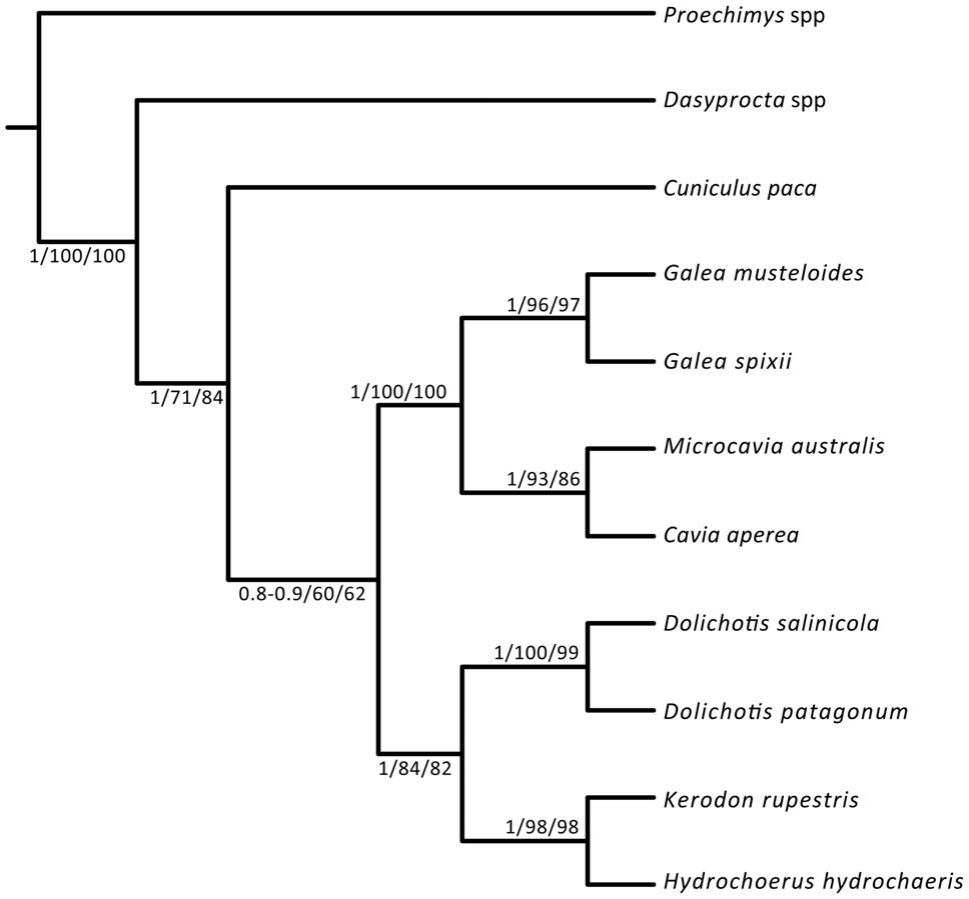
Phylogenetic relationships of extant-only taxa of Cavioidea with support values. This topology was retrieved for both the combined parsimony analysis and the Bayesian analysis of the molecular partition. For each node we indicate the posterior probability of the Bayesian analysis, the absolute bootstrap frequency of the parsimony analysis, and the absolute jackknife frequency of the parsimony analysis.

The phylogenetic analyses conducted here are highly congruent in terms of the retrieved topologies. The most parsimonious trees (MPTs) of the parsimony analysis of the combined dataset (including the morphological partition and all scored fossil taxa) differ in the interrelationship of some fossil forms of stem cavioids (e.g., *Eocardia* spp., *Schistomys*, *Matiamys*) and in the alternative positions of the fragmentary crown caviid taxon *Allocavia* (see Document S1.doc). The reduced strict consensus tree of the MPTs pruning *Allocavia* resolves the interrelationships of the three major lineages of Caviidae: Caviinae, Dolichotinae, and Hydrochoerinae (Figs. 1–2). This tree is identical in terms of the interrelationships of the living lineages of Cavioidea to the topology obtained in the Bayesian analysis of the molecular partition. Furthermore, if all fossils are excluded from the combined analysis, a single most parsimonious tree is found with identical topology as the one from the Bayesian analysis. Almost all nodes of this topology have high values of support (Fig. 3). In the Bayesian analysis all nodes had posterior probabilities that varied between 0.80 and 1.0. Similarly, if only the extant taxa are included in the parsimony analysis, the resampling support values (bootstrap, jackknife) of most nodes within Caviidae range between 80% and 100%, except for Caviinae that is 60% (Fig. 3).

**Figure 4:**
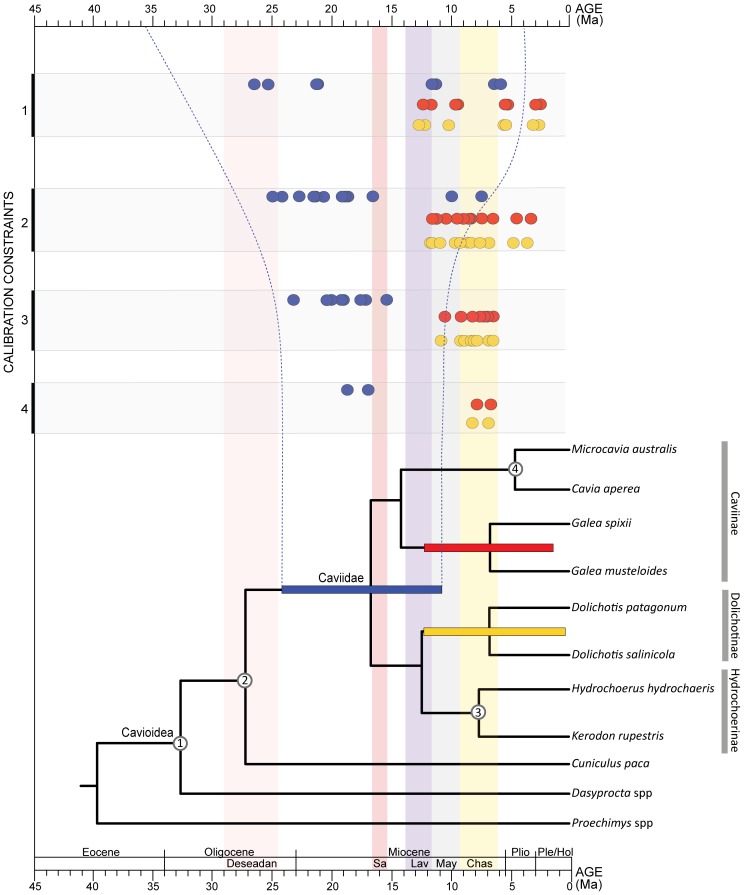
Phylogenetic hypothesis retrieved from the molecular dataset of extant cavioids obtained in BEAST. The time of divergence of each clade plotted in the tree is based on the mean estimates of the relaxed molecular clock using four calibration points. Colored bars in the tree represents the 95%HPD for the ages of Caviidae (blue), Dolichotinae (yellow), and the caviine *Galea* (red). Numbers on the tree indicate the calibration constraints used (see Calibrated Nodes). Dots on the left of the graphic indicate the mean age estimates for Caviidae (blue), Dolichotinae (yellow), and the caviine *Galea* (red) obtained with all possible combinations of one, two, three, and four calibrated nodes (using both normal and gamma prior distributions for calibrated nodes). Dotted blue lines represent the maximum breadth of the 95%HPD obtained for the age of Caviidae with different number of calibration constraints.

The calibration of the obtained phylogenetic hypotheses against time reveals the presence of three major phases in the evolutionary history of Cavioidea *s.s*. The first two of them are radiations exclusively revealed by fossil forms that are placed basally within Cavioidea *s.s*., prior to the origin of the crown-group Caviidae. These radiations are evident when the phylogenetic trees stemming from the parsimony analysis of the combined dataset are calibrated against geological time and characterize the early evolution of this group from the late Oligocene to the middle Miocene (Fig. 1). The third phase started in the middle to late Miocene and involves the diversification of the crown-group Caviidae (Figs. 1, 4). Given the presence of extant lineages of Caviidae, the timing of the diversification events of this third stage can be approached using both the fossil record of crown caviid and molecular clock estimates.

The evidence that leads to the recognition of these diversification events is summarized here in chronological order, discussing the timing inferred for these events and the evolutionary novelties that appeared at these times. The inferences made upon the fossil record evidence are presented first and the results of the molecular clock estimates are provided subsequently.

### Diversification Patterns Inferred from Fossils and Ghost Lineages

#### Phase 1: Radiation of basal Cavioidea *s.s*

The most ancient records of Cavioidea *s.s*. come from late Oligocene beds of Patagonia (Deseadan SALMA [South American Land Mammal Age]; 24.5–29 Ma; [61], [62]). Two taxa are known from this age: *Asteromys punctus* [49], [52], [63] and *Chubutomys simpsoni* [52]. Both taxa are only known by fragmentary specimens composed by mandibular fragments. The relatively limited fossil record of Deseadan age does not reveal by itself the presence of a radiation during this time. However, when the phylogenetic hypotheses obtained in the parsimony analysis of combined dataset are calibrated against the geological age of fossil taxa, a basal cavioid radiation is revealed by the presence of four ghost lineages that must have originated in the Deseadan age (in addition to the two species recorded for this age; Fig. 1). These four ghost lineages are present in all the most parsimonious trees (MPTs) and extend the minimum age of origin of the lineages leading to *Luantus initialis*, *Luantus minor*, *Chubutomys leucoreios*, and the clade formed by *Luantus propheticus* and more derived cavioids back to the late Oligocene (Deseadan SALMA). All these forms appear later in the fossil record, in early Miocene beds referred to the Colhuehuapian SALMA (18–20.5 Ma; [61], [62]). Therefore, the calibrated phylogenies extend the evolutionary origins of these lineages at least 4 Myr before their first appearance in the fossil record. The extent of these ghost lineages is caused by the relatively derived phylogenetic position of the Oligocene taxon *Chubutomys simpsoni* (and the paraphyly of the genus *Luantus*; Fig. 1). The character support for such a derived position of *Chubutomys simpsoni* is, however, moderately low (as it takes two extra steps to place it basally within Cavioidea *s.s*. and bootstrap and jackknife frequencies of the two nodes basal to *Chubutomys simpsoni* are approximately 40% and 60%; Fig. 2; see Document S1).

The discovery of a basal cavioid radiation in the late Oligocene contrasts with previous interpretations about the early evolution of Cavioidea *s.s.* [52], [55], [64]. These traditional hypotheses postulated that the early phases of cavioid evolution proceeded through gradual changes from the late Oligocene to the early Miocene, reflected in the progressive appearance of *Asteromys* and the species of *Luantus* in the fossil record. Our phylogenetic analysis rejects this interpretation, placing *Chubutomys simpsoni* deeply nested within basal cavioids given the presence of apomorphic features of its dentition (e.g., protohypsodont condition of the teeth and the absence of mesofossetid in early ontogenetic stages). This implies a previously undetected radiation of all basal lineages of Cavioidea *s.s.* that must have occurred at least by the late Oligocene. The optimization of character transformations in these basal nodes of Cavioidea *s.s*. indicates that major evolutionary novelties must have appeared in this late Oligocene radiation, including changes from a mesodont to a protohypsodont dentition and other dental modifications such as the appearance of cement and enamel discontinuities and the loss of fossettes/ids during ontogeny.

The ghost lineages provide a minimum estimate for the age of these basal nodes of Cavioidea *s.s.*, so it is possible that the actual diversification of this group predated the Deseadan SALMA (24.5–29 Ma). The pre-Deseadan record of fossil rodents in South America however provides relevant information to evaluate this possibility. The youngest pre-Deseadan rodent assemblage is known from La Cantera horizon of Central Patagonia (Upper Puesto Almendra Member, Sarmiento Formation; 29.5–31.1 Ma; [62]), which has yielded numerous rodent specimens but none belonging to Cavioidea *s.s*. (see also Node 2 in Calibrated Nodes). The La Cantera rodents include plesiomorphic taxa of Cavioidea (Dasyproctidae?) and representatives of Octodontoidea and Chinchilloidea [65]. Earlier faunal assemblages with fossil rodents from South America are limited to Tinguiririca in Chile (31.5–37 Ma; [29], [61]) and possibly the Santa Rosa fauna of Peru (estimated as late Eocene or Oligocene; [66]). The fossil rodents from these localities are also plesiomorphic forms of Cavioidea. Furthermore, the recently discovered rodent fauna from the Yahuarango Formation (ca. 41 Ma; middle Eocene; [28]) has yielded the oldest known South American rodents, which are basal forms of Caviomorpha that are not closely related to Cavioidea. The absence of Cavioidea *s.s*. in all these pre-Deseadan rodent faunas is compatible with the hypothesis of a basal radiation of this group close to or in the late Oligocene.

#### Phase 2: Radiation of euhypsodont cavioids

The euhypsodont condition represents the presence of teeth with continuous growth (lacking a root) and was one of the major evolutionary novelties in the history of Cavioidea *s.s*. The most ancient records of euhipsodont cavioids come from the early Miocene Santa Cruz Formation of Central Patagonia (Santacrucian SALMA; 15.7–16.5 Ma; [61], [67]–[69]). This unit has yielded thousands of specimens of cavioids, including the three youngest records of protohypsodont species (*L. toldensis*, *Phanomys mixtus*, *P*. *vetulus*) and the five oldest euhypsodont species of Cavioidea *s.s*. (*Eocardia montana*, *E. fissa*, *E. excavata*, *Schistomys erro*, *S. rollinsi*). The sudden appearance of multiple euhypsodont lineages at this time suggests the occurrence of an evolutionary radiation that not only includes the five above mentioned taxa but also three ghost lineages leading to slightly younger taxa of the Colloncuran SALMA (13,8–15.5 Ma; [70]): 1) *Matiamys elegans*, 2) *E. robertoi*, and 3) the clade formed by *E. robusta* and more derived cavioids (see Fig. 1). Thus, unlike the case of basal cavioids, the diversification of euhypsodont lineages is more evident in the number of species known from the Santa Cruz Formation than in the diversity inferred from ghost lineages. The morphological data provide strong support for the Santacrucian radiation of euhypsodont cavioids. The node formed by *Eocardia* and more derived cavioids and the node formed by *Phanomys* and more derived cavioids (see Fig. 1) have bootstrap and jackknife frequencies between 82% and 85% (Fig. 2; see Document S1). The Bremer support for these nodes is only moderate (Bremer  = 2) but forcing any of the pre-santacrucian cavioids (e.g., *L. propheticus*, *L. minor*) within this clade results in markedly suboptimal topologies (i.e., at least ten extra steps).

The exceptional Santacrucian fossil record and the sudden appearance of a high diversity of euhypsodont cavioids can be interpreted in two alternative ways. On the one hand, the fossil record could be actually capturing the early offshoots of a major radiation, characterized by the acquisition of a key evolutionary innovation (euhypsodonty), which has been interpreted as an adaptation to grazing. The acquisition of the euhypsodont condition may have been a critical innovation at this time given the major environmental changes recorded in Patagonia, which includes high volcanic activity related to the uplift of the Andes (Quechua phase; [71]), a change from grasslands and woodlands biomes, and a general drop in humidity and temperature [72], [73].

On the other hand, the exceptional Santacrucian fossil record could cause a Lagerstätten effect: the sudden first appearance of multiple lineages merely due to the presence of favorable conditions for fossilization. In this way, some lineages may have existed before the Santacrucian but were not captured by the fossil record just because the preservation potential of rodents in older sediments was unfavorable. A critical analysis of the cavioid fossil record in sediments older than the Santa Cruz Formation, however, lends support to the existence of a major radiation during the Santacrucian SALMA. The Pinturas Formation contains a sedimentary sequence that is slightly older than the Santa Cruz Formation, dated at 17.7 Ma [67]. The rodent fossil record in this unit is extremely rich, being the second-best record from the Oligocene-mid Miocene of Patagonia (after the Santacrucian). Out of the thousands of rodent specimens known from the Pinturas Formation, all the cavioid taxa are protohypsodont and there is no record of euhypsodont species in this large sample [55], [74]. Based on the high quality of the rodent fossil record of the Pinturas Formation, we believe that the sudden appearance of multiple euhypsodont species at the Santa Cruz Formation represents a true evolutionary radiation. Furthermore, the numerous cladogenetic events inferred to occur during the Santacrucian in the calibrated phylogeny (Fig. 1) are restricted to a very specific part of the cavioid tree, rather than distributed in different parts of the phylogeny. As noted by Calvin and Forey [75], such scenarios are more likely to result from a genuine radiation event. If the sudden diversity increase were due to a Lagerstätten effect, there would be no expectation to find all the new lineages clustered in one region of the phylogenetic tree.

#### Phase 3: Diversification of Caviidae

The oldest caviid known is *Prodolichotis pridiana* [76] from the La Venta section of Colombia (La Victoria and Villa Vieja formations; Laventan SALMA, middle Miocene, 11.8–13.5 Ma; [77]). This taxon has been traditionally referred to either Dolichotinae [76] or Caviinae [78] given the presence of derived similarities shared with these two lineages (e.g., presence of the nasolacrimal foramen in the maxilla). However, the morphological data used in the combined phylogenetic analysis presented here unequivocally places *P. pridiana* as the sister group of the third major lineage of caviids: Hydrochoerinae (including *Kerodon*; Fig. 1). This position is supported by the presence of two unambiguous synapomorphies (i.e., p4 with three lobes [character 45] and frontals not convex [character 59]). This position is however weakly supported given that *P. pridiana* can be positioned as the sister group of dolichotines with a single extra step or basally within Caviidae with two extra steps. Similarly, bootstrap and jackknife frequencies of the node joining *P. pridiana* and Hydrochoerinae range between 5% and 7% (Fig. 2; see Document S1).

Slightly older fossils recorded in deposits of Colloncuran age represent successive sister taxa of Caviidae (e.g., *Guiomys, Microcardiodon*; see Fig. 1) so that based on the available information *P. pridiana* is the most ancient Caviidae. The phylogenetic position of *P. pridiana* implies that the diversification of Caviidae in its three major lineages must have occurred, at least, by Laventan times (11.8–13.5 Ma; [69], [79]). After this time, the caviid fossil record has an extensive gap (of at least 2.7 Myr) until the late Miocene Arroyo Chasicó Formation of Central Argentina (Chasicoan SALMA; 6.1–9.07 Ma), in which derived specimens of the three modern lineages of caviids are recorded (i.e., Caviinae, Dolichotinae, Hydrochoerinae; see Calibrated Nodes). The only caviid remain that fill the extensive gap between the Laventan and Chasicoan records is a single isolated tooth from the Río Frias Formation (Mayoan SALMA) that is possibly related to basal hydrochoerines [80] (see Calibrated Nodes).

Therefore, the known fossil record lacks adequate information for thoroughly understanding the timing and radiation of modern lineages of crown caviids. The basic evolutionary pattern that can be inferred from the available fossil record is that: a) the initial split of Caviidae in three major lineages must have occurred at least 11.8–13.5 Ma (based on the Laventan record of *P. pridiana* and its phylogenetic position) and b) that the three modern and morphologically distinct lineages of Caviidae (Hydrochoerinae, Dolichotinae, Caviinae) were already present, abundant, and diverse about 6.1–9.07 Ma (based on the derived caviid fauna of late Miocene Arroyo Chasicó Formation and the ghost lineages that can be inferred from their phylogenetic positions; see Fig. 1).

### Molecular clock estimates

Understanding the tempo and mode of the diversification of Caviidae is especially interesting because this is the time when the three major living lineages acquired their distinctive morphologies, modes of life, body sizes, and social behaviors [35], [40], [41]. As noted above the fossil record lacks enough information to provide a complete picture of this critical time in the evolutionary history of Caviidae. The DNA sequences of extant caviids can provide insights on the timing of these evolutionary events that can be compared with the pattern emerging from fossil-based datings.

Previous molecular clock estimates on the time of diversification of Caviidae resulted in disparate dates [13], [20], [56], [59], ranging from slightly older (14 Ma) to markedly older (25 Ma) than the age of the oldest crown caviid (*P. pridiana*; 11.8–13.5 Ma). These estimates, however, were based on different DNA sequences and molecular clock methods so that it is difficult to compare their reliability and determine the causes of their differences.

Given the multiple nodes that are paleontologically dated by the phylogenetic study presented here (Fig. 1), we tested the influence of the choice of calibration constraints (see Calibrated Nodes), as well as the use of alternative prior probability distributions for the age of these calibrations on relaxed molecular clock estimates (see Calibrated Nodes and Table 1). The results of the 30 analyses conducted using different calibration constraints indicate a moderate to high level of rate heterogeneity depending on the calibration constraints used. The four independent MCMC runs conducted for each of the 30 analyses yielded extremely similar results in terms of the parameters of interest: topology and ages of diversification of the nodes of Caviidae (Tables 2, 3) and the ESS of the parameters of interest (ages of Caviidae and major lineages of caviids) are well above 200, indicating strong convergence. The extent of the 95% HPD of node ages depends on the prior distribution and the calibration constraints used, but all of them indicate a considerable degree of uncertainty in the relaxed molecular clock (see Table 3).

**Table 1:** Parameters used to set the prior distribution of ages in each of the 30 analyses of Bayesian relaxed molecular clock in BEAST v. 1.6.

Analysis	Distribution	Node 1	Node 2	Node 3	Node 4
1	Normal	34.5, 1.8	26.8, 1.4	7.6, 0.9	4.65, 0.4
2	Gamma	2, 2.85, 31.5	2.5, 1.05, 24.5	1.5, 1.85, 6.1	2, 0.76, 4
3	Normal	34.5, 1.8	26.8, 1.4	7.6, 0.9	TP
4	Gamma	2, 2.85, 31.5	2.5, 1.05, 24.5	1.5, 1.85, 6.1	TP
5	Normal	34.5, 1.8	26.8, 1.4	TP	4.65, 0.4
6	Gamma	2, 2.85, 31.5	2.5, 1.05, 24.5	TP	2, 0.76, 4
7	Normal	34.5, 1.8	TP	7.6, 0.9	4.65, 0.4
8	Gamma	2, 2.85, 31.5	TP	1.5, 1.85, 6.1	2, 0.76, 4
9	Normal	TP	26.8, 1.4	7.6, 0.9	4.65, 0.4
10	Gamma	TP	2.5, 1.05, 24.5	1.5, 1.85, 6.1	2, 0.76, 4
11	Normal	34.5, 1.8	26.8, 1.4	TP	TP
12	Gamma	2, 2.85, 31.5	2.5, 1.05, 24.5	TP	TP
13	Normal	34.5, 1.8	TP	7.6, 0.9	TP
14	Gamma	2, 2.85, 31.5	TP	1.5, 1.85, 6.1	TP
15	Normal	34.5, 1.8	TP	TP	4.65, 0.4
16	Gamma	2, 2.85, 31.5	TP	TP	2, 0.76, 4
17	Normal	TP	26.8, 1.4	7.6, 0.9	TP
18	Gamma	TP	2.5, 1.05, 24.5	1.5, 1.85, 6.1	TP
19	Normal	TP	26.8, 1.4	TP	4.65, 0.4
20	Gamma	TP	2.5, 1.05, 24.5	TP	2, 0.76, 4
21	Normal	TP	TP	7.6, 0.9	4.65, 0.4
22	Gamma	TP	TP	1.5, 1.85, 6.1	2, 0.76, 4
23	Normal	34.5, 1.8	TP	TP	TP
24	Gamma	2, 2.85, 31.5	TP	TP	TP
25	Normal	TP	26.8, 1.4	TP	TP
26	Gamma	TP	2.5, 1.05, 24.5	TP	TP
27	Normal	TP	TP	7.6, 0.9	TP
28	Gamma	TP	TP	1.5, 1.85, 6.1	TP
29	Normal	TP	TP	TP	4.65, 0.4
30	Gamma	TP	TP	TP	2, 0.76, 4

The parameters given for normal distributions are the mean and standard deviation, and the parameters given for the gamma distributions are the shape, scale, and offset. Uncalibrated nodes are marked with TP, representing the tree priors used in each analysis for the age of uncalibrated nodes.

**Table 2:** Mean age estimates for the age (in Ma) of nodes of interest based on the integration of the four independent MCMC runs made for each of the 30 different Bayesian relaxed molecular clock analyses.

Analysis	Node 1	Node 2	Node 3	Node 4	Caviidae	*Galea*	Dolichotinae	Caviomorpha
1	32.827	27.149	7.653	4.827	17.002	6.787	6.912	38.914
2	33.189	27.629	9.118	7.774	18.790	7.961	8.254	39.706
3	31.904	27.576	8.487	14.778	20.527	9.238	8.936	38.800
4	32.953	29.486	13.175	17.455	23.228	10.732	10.958	41.748
5	32.856	27.236	9.376	4.825	18.888	7.252	7.959	38.688
6	33.155	27.871	11.485	8.646	20.069	8.677	9.267	40.226
7	33.417	29.163	7.663	4.830	17.121	7.055	6.946	39.429
8	33.775	30.416	9.206	7.706	19.300	8.211	8.391	40.801
9	27.637	25.906	7.646	4.858	15.479	6.474	6.604	33.406
10	27.534	25.886	9.481	9.379	17.656	7.807	8.078	34.000
11	31.740	28.395	14.162	17.221	22.803	10.545	11.008	40.187
12	33.116	30.100	15.022	18.288	24.151	11.202	11.685	42.566
13	33.230	30.216	8.485	15.300	21.487	9.675	9.186	40.193
14	34.215	31.946	14.166	18.822	24.974	11.645	11.734	44.091
15	33.578	29.653	9.520	4.838	18.783	7.524	7.681	39.783
16	33.881	30.835	12.258	8.764	21.257	9.008	9.847	41.472
17	27.213	25.662	8.633	13.857	18.793	8.587	8.401	33.932
18	27.898	26.227	11.967	15.650	20.645	9.553	9.780	36.340
19	27.818	26.030	8.637	4.856	16.687	6.569	6.996	33.792
20	27.645	25.964	11.181	10.186	18.682	8.380	8.784	34.455
21	10.138	9.486	5.381	4.984	7.535	3.396	3.745	13.104
22	13.422	12.576	6.691	7.207	9.992	4.619	4.971	17.574
23	34.202	32.086	15.801	19.353	25.491	11.819	12.256	44.411
24	35.371	33.234	16.539	20.225	26.512	12.370	12.845	46.475
25	28.198	26.585	13.061	16.028	21.110	9.794	10.165	36.668
26	28.292	26.549	13.118	16.088	21.171	9.817	10.187	36.971
27	15.299	14.390	7.262	8.667	11.482	5.278	5.519	19.984
28	15.889	14.897	7.393	8.956	11.860	5.526	5.743	20.881
29	7.885	7.412	3.657	4.545	5.926	2.733	2.828	10.279
30	8.605	8.094	3.957	4.884	6.428	3.007	3.074	11.255

**Table 3:** 95% HPD for the age estimates (in Ma) for the age of nodes of interest based on the integration of the four independent MCMC runs made for each of the 30 different Bayesian relaxed molecular clock analyses.

Analysis	Node 1	Node 2	Node 3	Node 4	Caviidae	*Galea*	Dolichotinae	Caviomorpha
1	29.180; 36.444	24.459; 29.9	5.83; 9.398	4.06; 5.615	10.754; 24.426	1.898; 13.989	1.923; 13.052	30.084; 56.615
2	31.546; 36.537	24.897; 31.332	6.133; 13.268	4.167; 12.85	12.665; 24.774	2.523; 13.936	3.051; 14.323	31.941; 55.662
3	28.695; 35.426	25.068; 29.996	6.706; 10.278	9.206; 19.887	15.902; 25	4.926; 14.102	4.354; 13.632	29.99; 53.02
4	31.539; 35.671	25.911; 32.756	7.796; 17.106	11.445; 22.279	18.702; 27.279	6.075; 15.211	6.193; 15.034	32.603; 52.869
5	29.31; 36.378	24.589; 29.879	2.62; 17.211	4.029; 5.609	11.737; 25.654	1.804; 14.755	2.47; 15.429	29.842; 55.954
6	31.539; 36.631	24.911; 31.461	4.74; 17.554	4.243; 14.526	13.592; 25.843	3.233; 14.455	3.345; 15.301	32.042; 55.408
7	29.775; 36.991	20.128; 35.249	5.987; 9.44	4.071; 5.625	10.398; 25.537	2.242; 13.42	2.133; 12.999	30.373; 57.784
8	31.529; 38.647	22.379; 36.7	6.129; 13.499	4.244; 13.056	12.057; 26.645	3.085; 14.688	2.916; 14.149	32.103; 57.926
9	23.561; 33.413	23.091; 28.787	5.974; 9.404	4.086; 5.652	10.053; 22.688	2.108; 12.196	2.33; 12.17	24.718; 50.404
10	25.015; 31.572	24.588; 28.2	6.19; 12.983	4.587; 14.333	12.312; 22.27	3.332; 11.988	3.248; 12.427	26.113; 47.221
11	28.846; 34.762	26.048; 30.781	9.678; 18.495	12.478; 21.399	19.222; 26.165	6.613; 14.494	7.242; 15.112	31.535; 50.673
12	31.556; 35.979	26.411; 33.527	10.479; 19.463	13.319; 22.907	19.927; 28.298	7.322; 14.997	7.429; 16.051	33.074; 53.196
13	29.572; 36.924	24.795; 35.508	6.53; 10.368	4.818; 23.624	14.46; 28.756	1.513; 14.905	3.956; 14.968	31.085; 56.043
14	31.544; 39.448	27.418; 38.309	7.25; 18.451	12.03; 25.183	19.363; 31.041	7.023; 16.279	6.508; 16.863	33.285; 57.134
15	30.007; 37.257	21.362; 35.838	2.262; 17.345	4.064; 5.626	11.162; 28.277	1.955; 15.639	1.7; 15. 063	30.787; 58.697
16	31.571; 38.821	23.922; 37.678	5.168; 19.098	4.193; 15.32	13.229; 28.78	3.027; 14.852	3.426; 16	32.029; 57.839
17	23.372; 31.437	22.822; 28.516	6.757; 10.389	8.642; 18.701	14.439; 23.131	4.926; 12.466	4.848; 12.301	25.753; 45.434
18	25.089; 32.188	24.644; 29.021	7.469;15.241	10.539; 20.285	16.614; 24.517	5.484; 13.677	5.802; 13.951	27.338; 46.832
19	23.561; 33.545	23.211; 28.806	2.684; 15.915	4.061; 5.362	10.039; 23.771	1.677; 12.673	1.976; 13.607	24.99; 51.101
20	25.068; 31.815	24.578; 28.347	4.187; 16.294	4.464; 15.007	12.54; 23.479	3.69; 12.787	3.486; 13.487	26.287; 46.886
21	7.194; 14.999	6.739; 13.7	3.897; 7.325	4.242; 5.742	5.613; 10.335	2.149; 5.107	2.257; 5.97	8.278; 21.21
22	10.143; 18.069	9.724; 16.439	6.102; 8.079	5.121; 9.454	7.925; 12.831	3.035; 6.555	3.213; 7.019	11.841; 25.228
23	30.657; 37.779	27.712; 36.648	10.866; 20.686	14.169; 24.431	20.65; 30.321	7.904; 16.012	8.049; 16.821	33.722; 56.359
24	31.588; 42.768	27.858; 42.184	10.94; 23.897	13.961; 28.007	20.7; 35.162	7.96; 17.932	7.728; 19.399	34.242; 61.153
25	24.731; 32.124	23.836; 29.301	9.216; 17.322	11.644; 20.078	17.362; 24.711	6.583; 13.141	6.57; 13.959	27.873; 46.461
26	25.372; 32.619	24.584; 29.716	2.635; 17.233	10.55; 21.112	16.943; 26.172	4.998; 14.207	3.793; 15.453	28.023; 47.119
27	9.935; 21.803	9.269; 20.207	5.515; 9.12	5.291; 12.568	7.75; 15.732	2.968; 7.914	3.284; 8.18	12.09; 29.888
28	10.781; 25.699	10.215; 23.521	6.1; 10.682	5.426; 15.611	8.293; 18.455	3.233; 9.114	3.376; 9.688	12.766; 34.501
29	5.772; 10.499	5.486; 9.784	2.438; 5.191	3.725; 5.318	4.491; 7.609	1.787; 3.895	1.75; 4.193	6.937; 14.394
30	6.147; 12.885	5.815; 11.973	2.474; 6.438	4.025; 6.644	4.812; 9.335	1.892; 4.79	1.628; 5.454	7.334; 17.43

The lower and upper bound of the 95% HPD are given for each analysis separated by a semicolon.

The results of the analyses involving different number of calibration constraints and their prior probability distributions are summarized below for the time of diversification of Caviidae and the diversification of the major modern lineages of Caviidae (see also Tables 2 and 3).

#### Four calibration constraints

Owing to the incorporation of fossils in the phylogeny, these are the most tightly constrained analyses of the diversification timing of Caviidae (Fig. 4). The estimate of the time of diversification of Caviidae using gamma prior distribution yields a mean estimate of 18.8 Ma (leftmost blue circle in row 4 of Fig. 4; Table 2) and a 95% HPD ranging between 12.66 and 24.77 Ma (Table 3) in the four independent MCMC runs (ESS = 480). As in most analyses, when a normal prior distribution is used the estimates are slightly younger, yielding a mean age of 17 Ma (rightmost blue circle in row 4 of Fig. 4; see Table 2) and 95% HPD ranging from 10.75 to 24.43 Ma (Table 3). In both cases, the age of the oldest fossil caviid (*P. pridiana*; Laventan age; purple column in Fig. 4) is younger than the mean age estimate but falls within the 95% HPD (blue bar on tree of Fig. 4; Table 3).

The initial diversification of Caviinae and the split between Hydrochoerinae and Dolichotinae are inferred to occur two to five million years after the initial diversification of Caviidae, with mean gamma estimates of 16.37 and 14.42 Ma for these two clades and normal estimates of 14.08 and 12.30 Ma (Fig. 4). An interesting pattern in these estimates is the coincident time of diversification of three major modern and morphologically distinct linages of Caviidae (Fig. 4): Dolichotinae, Hydrochoerinae (node 3), and the caviine lineage leading to the genus *Galea*. The age of one of these nodes was constrained by a prior distributions (node 3; see Calibrated Nodes) but the ages retrieved for the diversification of Dolichotinae (yellow circles in row 4 of Fig. 4) and *Galea* (red circles in row 4 of Fig. 4) are highly congruent, with mean ages of 6.91 and 6.79 (normal distribution of calibration constraints; see Table 2) and 8.25 and 7.96 (gamma distribution of calibration constraints; see Table 2). The estimated ages of these nodes lie within the range of ages of the Chasicoan sediments, in which the oldest members of caviines, dolichotines, and hydrochoerines are recorded.

#### Three calibration constraints

These analyses sequentially exclude one of the four calibration constraints (see Calibrated Nodes and analyses 3 to 10 in Table 1), to test their influence in inferring the time of diversification of Caviidae. The estimate of the initial diversification of Caviidae excluding node 1, node 2, or node 3 (central blue circles in row 3 of Fig. 4) yield similar results as the estimate with four calibration points (mean age ranging between 15.48 and 20.01 Ma and 10.05–26.64 Ma 95% HPD; see Tables 2, 3). However, when the caviine calibration constraint (node 4) is excluded, the retrieved age are markedly older (leftmost blue circles in row 3 of Fig. 4), and the age of the oldest fossil caviid (*Prodolichotis pridiana*) lies outside the 95% HPD (15.9–27.28 Ma; see Table 3).

As with the estimates of the diversification of Caviidae, the estimates on the time of diversification of the modern linages of Caviidae are highly sensitive to the exclusion of node 4 from the calibration set. Excluding any of the other three calibration constraints (nodes 1, 2, or 3) the mean ages of these nodes fall within the Chasicoan SALMA (see Table 2). However, when node 4 is excluded from the calibration set, the estimated age of these nodes is older. This is especially marked when a gamma distribution is used, yielding mean age estimates that predate the Chasicoan age (leftmost red and yellow circles in row 3 of Fig. 4; Table 2). Interestingly, the mean ages inferred for node 4 (*Microcavia*+*Cavia*) when this node is not calibrated is remarkably old, with mean ages of 14.78 and 17.45 Ma and 95% HPD (Table 3) lying outside the Chasicoan (significantly predating the appearance of derived caviines in the fossil record) This suggests that either this caviine lineage has a higher evolutionary rate than the other caviids or that its fossil record failed to capture more than 60% of their evolutionary history. These two hypotheses are plausible explanations but more data and research is needed for testing them.

#### Two calibration constraints

These analyses retrieved disparate results on the time of diversification of Caviidae (see analyses 11 through 22 in Table 1). Out of the 12 conducted analyses, six of them yielded 95% HPD age estimates for the initial diversification of Caviidae that are older than the first fossil caviid and one of them yielded 95% HPD age estimates that are younger than the first fossil caviid (see Table 3). The six analyses that excluded the caviine calibration constraint (node 4 in Fig. 4; see Calibrated Nodes) yielded the oldest age estimates for the initial diversification of caviids (Table 2). The inclusion of the caviine calibration point (node 4) in combination with the calibration of the basal nodes of Cavioidea (node 1 or node 2) yielded mean age estimates (central blue circles in row 2 of Fig. 4) that are similar to those of the analysis with four constrained nodes (Table 2). The 95% HPD of these analyses included the age of appearance of fossil caviids (Table 3). However, when the two calibration constraints were node 4 and node 3, the estimated mean age and 95% HPD resulted exceedingly young (Table 3) in comparison with the caviid fossil record (mean age estimates ranging between 7.53 and 9.99 Ma, using a normal and gamma distribution; rightmost blue circles in row 2 of Fig. 4; Table 2).

The conducted analyses also retrieved highly variable results on the time of diversification of the major modern lineages of Caviidae mentioned above (Fig. 4). As for the estimates on the age of Caviidae, when the caviine calibration (node 4) was excluded, the inferred ages of several nodes are markedly older, with most mean estimates of most nodes dating over 9.5 Ma (leftmost red and yellow circles in row 2 of Fig. 4; Table 2). Again, this is particularly noticeable when a gamma distribution is used in the prior probability of the calibration constraints. When node 4 is included in the calibration set in combination with the basal most calibration constraints (nodes 1 or 2) the estimates on the diversification ages of the modern lineages of Caviidae retrieved mean ages that fall within the Chasicoan SALMA (central yellow and red circles in row 2 of Fig. 4; Table 2). The use of only the two most recent constraints (nodes 3 and 4) yields mean ages that are much younger than the Chasicoan age (rightmost yellow and red circles in row 2 of Fig. 4; see Table 2). In most of these estimates, however, the 95% HPD is notably large and extends for more than 10 Myr (for both normal and gamma prior distributions; see Table 3) covering from the Pleistocene to the Miocene (thus completely including the Chasicoan age).

#### One calibration constraint

These analyses also retrieved disparate results on the timing of caviid evolution (analyses 23 through 30 in Table 1). All analyses that used the calibration of the two basal nodes of Cavioidea (node 1 and node 2; see Calibrated Nodes) retrieved remarkably old mean ages (leftmost blue circles in row 1 of Fig. 4; Table 2) with 95% HPD that are older than the first appearance of caviids in the fossil record (Table 3). Conversely, when node 4 is used as the single calibration point, the 95% HPD for the time of the initial diversification of caviids is younger than the first appearance of this group in the fossil record (rightmost blue circles in row 1 of Fig. 4; see Table 3). Finally, node 3 is the only calibration point that retrieves mean ages close to the age of the most ancient caviid *P. pridiana* (central blue circles in row 1 of Fig. 4; Table 2) and 95% HPD that includes this age (Table 3).

The estimates on the time of diversification of the major modern lineages of Caviidae are also highly sensitive to the choice of a single calibration constraint. When any of the two basal calibrations were used (node 1 and node 2), the mean ages of the Dolichotinae and *Galea* nodes are notably old (ranging from 9.79 to 12.84 Ma with both normal and gamma prior distributions; four leftmost red and yellow circles in row 1 of Fig. 4; Table 2). Furthermore, using these calibrations, the 95% HPD of the age of two other modern lineages of caviids (nodes 3 and 4) are in most cases exceedingly old (i.e., older than the Chasicoan SALMA; Table 3). Conversely, when the caviine age (node 4) is used as the only calibration constraint, the mean ages and the 95% HPD for the time of diversification of modern caviid lineages are younger than their first appearance in the fossil record at the Chasicoan SALMA (rightmost yellow and red circles in row 1 of Fig. 4; Table 3). Finally, as in the case of the age of Caviidae, when node 3 is the only calibration constraint the mean ages lie close to the upper bound of the Chasicoan age (5.3 to 5.7 Ma; see Fig. 4 and Table 2) and the 95% HPD of all the modern lineages includes the Chasicoan age (Table 3).

#### Summary of molecular clock estimates

**Figure 5:**
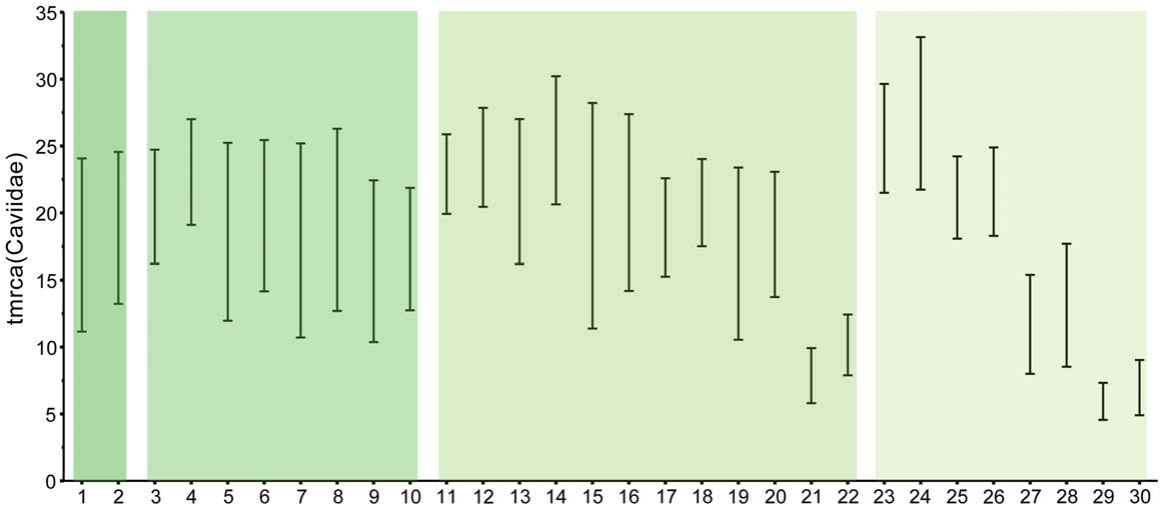
95%HPD of age estimates for the time of diversification of Caviidae as estimated for each of the 30 different Bayesian relaxed molecular clock analyses conducted in this study.

These results indicate a considerable degree of rate heterogeneity among groups of Cavioidea. The two oldest and most basal calibration constraints (node 1 and node 2; see Calibrated Nodes) lead to inferences of a much slower rate of evolution than when the caviine calibration constraint (node 4) is used, and rate estimates derived from calibrations of node 3 are intermediate between these two extremes. Consequently, estimates on the age of Caviidae and on the radiation of the major modern lineages of caviids based only on the basal calibrations (or basal nodes and node 3) are much older than those retrieved when node 4 (or node 4 and node 3) is used for calibrating the relaxed molecular clock (Fig. 4). This sensitivity to the choice of calibration constraint is also reflected in the 95% HPD that show variable results depending on the calibrate node/s. These distributions, however, converge to a similar result as the number of calibrated nodes increases, as exemplified for the 95% HPD of the age of Caviidae (Fig. 5). Based on these results, the use of four calibration points better accommodates the rate heterogeneity of the group as a whole, because these bracket the nodes of interest (Caviidae and the principal modern lineages of Caviidae). Therefore, we will discuss below the molecular clock inferences based on four calibration points (row 4 of Fig. 4) in terms of their agreement with the diversification pattern inferred from the fossil record of early caviids.

## Discussion

**Figure 6:**
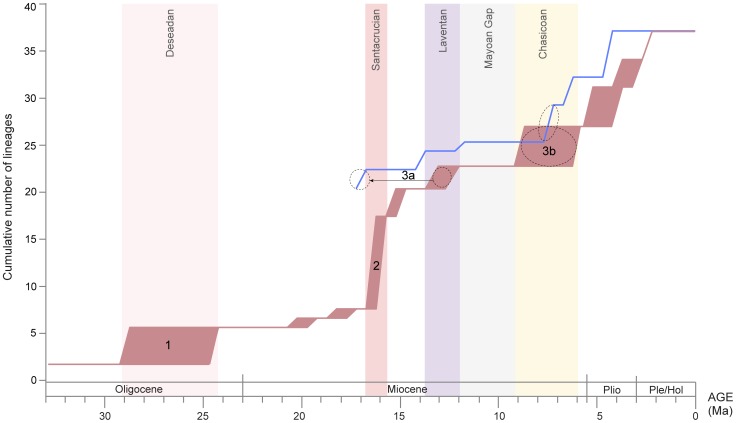
Diversification plot of cumulative number of cavioid lineages (leading to both extinct and extant species) against geological time. The red curve represents the number of lineages based on the simultaneous analysis of morphological and molecular data of both extinct and extant taxa (as shown in Fig. 4). The uncertainty in the geological age of each fossil creates a maximum and minimum times of divergence and is represented by the breadth of the diversification events on the red curve. The blue line represents the timing of diversification events of crown caviid lineages based on mean age estimates of the molecular clock estimates. The most relevant SALMAs are highlighted and the numbers 1, 2, 3a, and 3b represents the Deseadan radiation (1), the Santacrucian radiation (2), the initial diversification of Caviidae (3a), and the diversification of modern and morphologically well differentiated lineages of caviids during the Chasicoan (3b). The black arrow marks the discrepancy between the timing of the initial diversification of Caviidae based on the paleontological record (red curve) and the molecular clock estimates (blue curve).

The integration of the morphological and molecular data in the phylogenetic analyses conducted here and the temporal information from the fossil record and molecular clock estimates reveal the basic diversification patterns during the early evolution of Cavioidea *sensu stricto* and the crown-group Caviidae. The cumulative number of lineages (counting those leading to both extinct and living taxa) plotted across time reveals the diversification events of this group inferred from fossils and the molecular clock (Fig. 6). This plot contrasts the timing of each diversification event inferred from the age of first appearance of fossil taxa (red curve in Fig. 6) and from the molecular clock estimates (blue curve in Fig. 6). This diversification plot highlights the agreement and discrepancies of the results presented in this study.

### Diversification of Cavioidea *sensu stricto*

The morphological data of extinct species and their stratigraphic record document two early diversification phases: the Deseadan and the Santacrucian radiations (1 and 2 in Fig. 6). These radiations can only be inferred from the fossil record, as the only lineage that survived the Miocene was the one leading to Caviidae.

As noted above, the Deseadan radiation (1 in Fig. 6) involves the first record of Cavioidea *s.s.* (24.5–29 Ma; late Oligocene; Deseadan SALMA) and is recognized mainly by the four ghost lineages of forms that appear later in the fossil record (early Miocene; Colhuehuapian SALMA). Only two species are known up to date from this age, which provide the minimum estimate for the age of basal nodes of Cavioidea *s.s*. Although a more gradual diversification of this group might have occurred before the Deseadan SALMA, the older rodent assemblages at Tinguiririca and La Cantera lack members of this clade. An initial radiation during the late Oligocene fits the available fossil data and the morphological phylogeny.

The Santacrucian radiation (2 in Fig. 6) is the earliest record of euhypsodont cavioids (15.7–16.5 Ma; early Miocene; Santacrucian SALMA). It is mainly recognizable from the Patagonian fossils of a remarkably abundant and diverse rodent fauna. Fossils offer direct evidence of multiple euhypsodont lineages present at this time, unlike the Deseadan event, which was mainly inferred by the evidence for ghost lineages. However, as in that case, stratigraphic evidence–the lack of euhypsodont taxa in the well-sampled rodent faunas of older sediments of Patagonia (e.g., “Pinturan” or Colhuehuapian ages)–identifies this as a discrete evolutionary event rather than an artificial aggregation of diversity owing to taphonomic processes (e.g., Lagerstätten effect).

### Diversification of Caviidae

The diversification of the crown-group Caviidae (phase 3 in Fig. 6) involved the appearance of lineages that survived until today. Both the fossil record (red curve in Fig. 6) and inferences from the molecular clock (blue curve in Fig. 6) help to illuminate this event. Three distinct periods are identifiable: the initial split of Caviidae in three major lineages (3a in Fig. 6), followed by an obscure period with poor fossil record (Mayoan gap; Fig. 6), and then by the diversification and establishment of the morphologically and ecologically distinct modern lineages of Caviidae (3b in Fig. 6). Whereas the molecular clock estimates differ from the information provided by the fossil record regarding the timing of the initial split of Caviidae (3a), both sources of information agree on the timing and characteristics of the two subsequent periods (Mayoan gap and 3b; see Fig. 6).

### Initial split of Caviidae

This event involved the split between the caviine, dolichotine, and hydrochoerine lineages. The minimum age of this event is dated stratigraphically by the appearance of *Prodolichotis pridiana* (11.8–13.5 Ma; middle Miocene; Laventan SALMA), as shown by the diversification plot (3a in red curve of Fig. 6). Molecular clock inferences, however, yielded a mean age estimate that predates this age by 3.5 to 7 million years (17 and 18.8 Ma using normal and gamma priors, respectively), represented by the shifted position of this event in the diversification curve of molecular clock dates (3a in blue curve of Fig. 6). The timing of the initial diversification of Caviidae is therefore the major disagreement between the molecular dates and the fossil record.

The uncertainty on the relaxed molecular clock estimates is however relatively large, and the 95% HPD (normal  = 10.75–24.43 Ma and gamma  = 12.66–24.77 Ma; see Table 3) includes the Laventan age. On the other hand, there is some phylogenetic uncertainty related to the alternative (suboptimal) positions that some fossil taxa can take based on their morphology. *Prodolichotis pridiana* can be placed as the sister taxon of all caviids with two extra steps (pushing forward the time of diversification of Caviidae and increasing the discrepancy with molecular clock estimates). Alternatively, the Colloncuran *Guiomys unica* can be placed within Caviidae with a single extra step (pushing back the time of diversification of Caviidae to the Colloncuran and decreasing the discrepancy with the molecular clock estimates).

The molecular clock estimates, therefore, indicate there is a high probability that the fossil record is currently missing the first few million years of caviid evolution, although the breadth of the 95% HPD and the possible alternative positions in slightly suboptimal trees of some key fossils indicates the two sources of information are not yielding entirely incompatible estimates. Further studies on some of these fossils (e.g., *Prodolichotis pridiana*) and new remains of incompletely known taxa (e.g., *Guiomys unica*), as well as new sequences to base molecular clock estimates on a more extensive dataset are all necessary steps to solve this apparent discrepancy (see also *Reconciling molecular dating with the fossil record*).

### Mayoan Gap

After the Laventan age, the caviid fossil record has an extensive gap that stretches over the Mayoan SALMA (Fig. 1) and extends until the late Miocene. During the late Miocene (Chasicoan-Huayquerian SALMAs; see Calibrated Nodes) the oldest and already well differentiated members of the three modern clades of Caviidae (Caviinae, Dolichotinae, Hydrochoerinae) appear in the fossil record. Only two isolated cavioid teeth are known from the Mayoan SALMA (Río Frias Formation) and they have been recently interpreted [80] either as a close relative of the crown-group Caviidae (*Microcardiodon*) or as a crown caviid that bears plesiomorphic dental characters (e.g., convex walls of the prisms, relatively short accessory fissures) that resemble the condition of basal hydrochoerines (i.e., *Cardiomys* or *Kerodon*) but is markedly different from the highly modified condition of more derived hydrochoerines from the late Miocene (e.g., *Cardiatherium*). The scarce available evidence suggests that the initial diversification of Caviidae (in Laventan times or earlier; 3a in Fig. 6) was an event temporally separated from the acquisition of the derived morphology and the radiation of modern lineages of caviids (in Chasicoan times; 3b in Fig. 6) by the Mayoan gap (Fig. 6). This scenario predicts that if additional Mayoan caviid fossils are found they are likely to represent basal forms of caviids or caviid outgroups rather than early members of derived and morphologically differentiated clades of Caviidae.

### Diversification of modern caviid lineages

As noted above, the Arroyo Chasicó Formation records multiple lineages of derived caviids [53], which represent the earliest record of the clearly distinct body plans, body size ranges, apomorphic morphological traits, and probably the ecological roles of modern lineages of Caviidae (e.g., Dolichotinae, Hydrochoerinae, Caviinae). The Chasicoan radiation has been recognized on the basis of the diverse fossil fauna recorded in central Argentina [53], [81], a pattern that is corroborated here based on the combined phylogenetic analysis and the calibration of these events stratigraphically (Fig. 1) as well as the molecular clock estimates (Fig. 4). The radiation of modern and well differentiated caviid lineages (3b in Fig. 6) is inferred to occur during the Chasicoan SALMA both by the paleontological dates and by the mean age estimates using a relaxed molecular clock. The timing of this event in the cumulative diversity curve based on fossil dates (3b of red curve in Fig. 6) largely coincides with the cumulative diversity curve inferred from molecular clock estimates (3b of blue curve in Fig. 6). The sudden appearance of multiple lineages with markedly different morphological and ecological characteristics suggests the Chasicoan was indeed a key stage in caviid evolution, which resulted in the establishment of the distinct body plans of extant forms through an adaptive radiation.

### Reconciling molecular dating with the fossil record

Discrepancies of paleontological and molecular dates are relatively common and often, molecular clock estimates yield ages that are much older than the first appearance of taxa in the fossil record. This has lead to heated debates on the relative merits of both approaches [1], [3], [5], [82], [83]. Such differences can be attributed to deficiencies of both the methods of molecular clock estimation and/or to the quality of the fossil record.

### Molecular clock: potential biases

Several authors have noted molecular clock methods can suffer from biases that produce exceedingly old divergence dates [5], [7], [84]–[86]. These include the presence of extreme rate heterogeneity that cannot be accounted by current methods (e.g., gamma distribution, invariant sites), such as the possible inadequacy use of homogeneous Markov models of nucleotide evolution due to the existence of heterotachy [87]–[89], which would affect branch length estimation and therefore molecular clock estimates. Although the use of relaxed clocks has been proposed as a way to at least ameliorate some of these problems [60] and current methods provide molecular clock estimates with credibility intervals, the extent of the above noted problems in empirical datasets is difficult to assess.

It is worth noting that the credibility intervals of relaxed Bayesian molecular clock estimates (95% HPD) can be of substantial duration, and many times they do overlap with the first appearance datum of fossil taxa, as in the case of the initial diversification of Caviidae and the Laventan age. In many instances, therefore, the discrepancies between the molecular clock and the fossil record disappear when then 95% HPD are considered.

One of the interesting outcomes of the 30 different molecular clock analyses conducted here is the identification of the sensitivity of the results to the inclusion or exclusion of node 4 (*Microcavia*+*Cavia*). A striking difference exists between the age inferred by the molecular clock when this node is not calibrated and the first appearance of members of this clade in the fossil record. As noted above, the molecular clock estimates are likely too old and suggest the fossil record of caviine rodents would be missing 60% of its evolution. We suggest it is more likely this caviine lineage has a higher evolutionary rate in comparison with other cavioid rodents (at least for these genes). Caviines have a reduced body size (and related life-history traits such as shorter generation times) in comparison with other cavioid rodents (e.g., dolichotines, hydrochoerines), providing another case of correlation between high evolutionary rates and small body size if this hypothesis is correct. More data are needed to test this correlation, as well as to provide reliable molecular clock estimates for Caviinae. New data must include both a more extensive taxon sampling among caviines for these sequences and further studies on fossil caviines to provide alternative calibration points within this clade.

### The fossil record: potential biases

The fossil record is inherently incomplete and can certainly fail to capture representatives of two lineages soon after they split from their most recent common ancestor, underestimating the age of a clade's initial divergence [90]. Most importantly, in addition to being incomplete, the fossil record also has well-recognized biases that can systematically fail to capture the earliest members of the lineage of interest (selective incompleteness). The most obvious biases of the fossil record that explain the poor record of some lineages are those related to intrinsic biological causes (e.g. low fossilization potential of their body parts, small body size) or related to extrinsic causes due to the incompleteness of the sedimentary record. The latter include the absence/rarity of sedimentary rocks from a given period of time (i.e. non-depositional hiatuses), from the geographic region where the lineage might have originated (i.e. biogeographical biases), and/or from the ecosystem where the organisms lived (i.e. environments with low sedimentary rates). It is therefore critical to consider these possible biases when divergence dates from the fossil record are being compared with molecular clock estimates.

The case of the diversification of Caviidae allows exploring some of these potential biases of the fossil record, as their record is remarkably good in comparison with other terrestrial vertebrates from the Cenozoic of South America. The molecular clock mean age estimates on the initial diversification of Caviidae is up to seven million years older than the date documented by fossils, placing the basal split of Caviidae at the “Pinturan”/Santacrucian boundary (3a of blue curve in Fig. 6). The fossil record of cavioid rodents during these ages is remarkably abundant, including hundreds of cavioid specimens representing at least ten different species. This high diversity and abundance of cavioid actually represents the basal euhypsodont radiation (2 in Fig. 6) and none of these fossils can be identified as members of the crown-group Caviidae.

**Figure 7:**
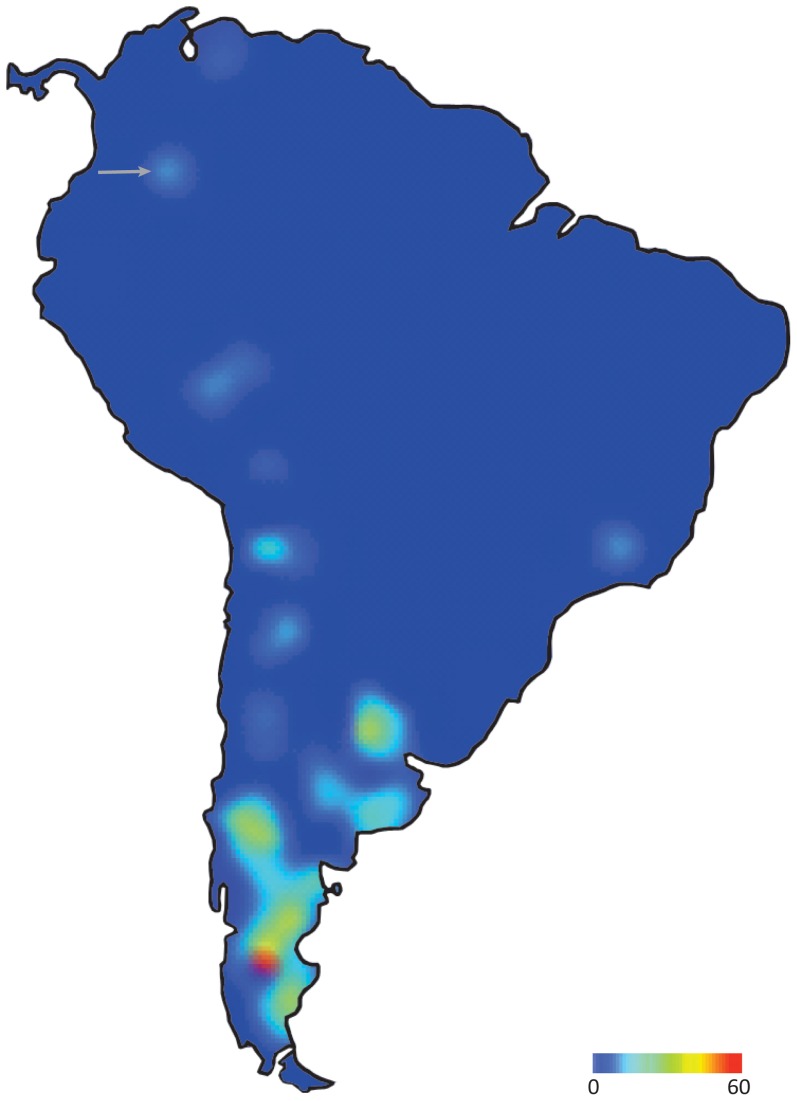
Species diversity map of fossil rodents in all known Oligocene-Pleistocene fossiliferous localities across South America. The number of valid species known for each region has been compiled and color coded. Blue colored regions represent the absence of rodent remains and warmer colors represent increasing number of species (taxonomic diversity) of fossil rodents. The red colored region highlights the highest species diversity of fossil rodents recorded in the “Pinturan”/Santacrucian fossiliferous localities of southern South America (Patagonia). Grey arrow indicates the rodent record of Laventan age in Colombia, where the oldest Caviidae (*P. pridiana*) is recorded.

The well sampled “Pinturan”/Santacrucian cavioid fauna could be used as evidence rejecting the molecular clock estimates on the time of diversification of Caviidae. However, the adequacy of the cavioid fossil record to reject the molecular dates should be evaluated considering its completeness not only through time but also through space. Mapping the geographic distribution and the number of fossil species of rodents during the Oligocene-Pleistocene in South America reveals an uneven geographic coverage of the fossil record in this continent (Fig. 7). The highly fossiliferous “Pinturan”/Santacrucian deposits are restricted to southern South America (Patagonia; southernmost red/yellow areas in Fig. 7) and therefore the high degree of certainty that crown caviids were absent from Patagonia during “Pinturan”/Santacrucian may not be valid for all of South America. Interestingly, the oldest fossil caviid (*P. pridiana*) comes from northern South America (Colombia; grey arrow in Fig. 7), a region completely lacking a pre-Laventan rodent fossil record. If the crown-group Caviidae actually originated at low latitudes in South America about 18 Ma, the available fossil record could not reflect this evolutionary event, as all the fossiliferous rocks from this age are restricted to the southern South America (Patagonia; see Fig. 7).

This biogeographic bias actually occurs for the entire rodent fossil record of the Cenozoic of South America, which despite being remarkably abundant is heavily biased towards fossils found at higher latitudes (Fig. 7). This geographic bias of the terrestrial fossil record of the Cenozoic of South America is extensive, at least to some degree, to all groups of fossil mammals and therefore should be considered when studying the evolutionary events of multiple fossil groups. Our current knowledge on the diversity of rodent fossils is remarkably good in mid- to high latitudes of South America. Numerous species are known from highly fossiliferous and extensive outcrops located in the southern half of South America (especially in Patagonia). These geological units have been known for over a century and intensive collecting efforts have been conducted on fossil mammals since the initial discovery of these outcrops [91]. In contrast, the rock availability and exposures in the Neotropics is much more restricted and collecting efforts conducted at higher latitudes of South America [92]–[96] have not been as intensive as in Patagonia.

As stated above, an incomplete fossil record is caused not only by non-depositional hiatuses but also involves the incomplete representation of the geographical distribution of a clade. We have no doubts that the fossil record can and should be used to refine and clarify the chronology of diversification events. However, its limitations and biases need to be borne in mind, particularly when we have to evaluate its conflict with other methodologies, such as a molecular clock. Ignoring the limitations and uncertainties of the fossil record could be as dangerous as ignoring the uncertainties associated with molecular clock estimates (e.g., 95% HPD of relaxed Bayesian clock estimates).

## Conclusions

Estimating the divergence time of clades based on phylogenetic studies is the area of most intense interaction (and conflict) between paleontology (providing fossils with dates) and molecular systematics (providing molecular clock estimates), as both areas provide information for understanding the tempo and mode of the evolution of a group. Recent efforts and progress have been made to incorporate different kinds of uncertainties to both molecular clock methods [60] and paleontological dating of divergence times [97]. One of these is the inherent uncertainty of the fossil record that needs to be considered for selecting calibration constraints, which current methods allow to accommodate [98]. We explored here two other important uncertainties that need to be considered when contrasting molecular clock estimates with paleontological dating of divergence times are: the uncertainties derived from molecular clock estimates (95% HPD in Bayesian methods) and the critical evaluation of the adequacy of the available fossil record (especially when the divergence dates estimated from both sources do not match).

Our study integrates morphological and molecular data gathered from extinct and extant taxa into a phylogenetic analysis of Cavioidea *s.s.* These analyses result in a global picture on the evolutionary history of Cavioidea *s.s*., including the origins and diversification of Caviidae, one of the most remarkably disparate lineages of living rodents. Three major evolutionary phases are recognized in the history of the group. The first two were radiations of basal forms that acquired the dental hallmarks of the groups: the appearance of protohypsodont forms in the Deseadan radiation of Cavioidea *s.s*. and of euhypsodont cavioids in the Santacrucian radiation, the evidence coming mostly from the Patagonian fossil record. The third phase was the diversification of Caviidae, which seems to have occurred in two temporally discrete episodes, the initial split of the group in three major lineages and the subsequent diversification of its modern clades, which are highly differentiated morphologically and ecologically.

A general agreement exists on the divergence dates estimated from molecular data and the fossil record. Molecular clock estimates places the origin of most modern lineages of caviids close to the Chasicoan, which coincides with the earliest appearance in the fossil record of the modern caviid lineages, which are characterized by remarkably distinct body plans, body size ranges, and ecological roles (dolichotines, hydrochoerines, and caviines).

However, the timing of the initial diversification of Caviidae was detected as the major discrepancy. The initial split of Caviidae is inferred to occur at Laventan times by paleontological evidence or perhaps as much as 7 million years earlier using a relaxed molecular clock. However, the uncertainties of the paleontological and molecular estimates reveal that more data is needed to solve this apparent conflict between the fossil record and the molecular clock.

From a paleontological point of view, a more extensive knowledge of pre-Laventan faunas are critical to clarify the time and place of the initial diversification of Caviidae. Although the record of pre-Laventan faunas (i.e., Friasian, Colloncuran) is geographically extensive, rodent faunas from these ages are all restricted to the southern half of South America (Chile, Argentina, and Bolivia), limiting our ability to localize the group's origin.

Finally, increasing the amount of molecular data (taxon and gene sampling) is also needed to achieve a more robust phylogenetic framework for caviid evolution and to generate more robust molecular clock inferences. The prospective integration of new sources of evidence into an integrated approach will be unavoidable steps for understanding the evolutionary history of Cavioidea *s.s.*

## Methods

### Combined Parsimony Analysis (morphology + DNA)

#### Morphological data

The morphological dataset was expanded from a recent study [48], [49] through the incorporation of six crown caviid taxa (four fossils, two extant) and ten new morphological characters. The morphological dataset included fossil and living representatives of Cavioidea *s.s.* that were selected as the ingroup. The dataset include all the known stem-group fossil taxa (“eocardiids”), at least one representative of each extant genus of Caviidae, and nine extinct species of caviids (see Document S2). Outgroup taxa included representative species of Dasyproctidae, Cuniculidae, and Echimyidae, the latter of which was used to root the topologies (see Document S2). The character sampling is based on 69 craneo-mandibular and 27 dental characters (see Document S3).

#### Molecular data

The DNA sequences of extant caviid species were gathered from GenBank for two nuclear and two mitochondrial genes: exon 10 of growth hormone receptor (*Ghr*), intron 1 of transthyretin (*Tth*), 12 subunit ribosomal RNA (*12s*), and cytochrome b (*cyb*). Sequences of these genes were available for nine extant representatives of Caviidae and the three outgroup taxa (see Document S2 for GenBank accession numbers). Sequences of two of the outgroup taxa (*Proechimys* and *Dasyprocta*) have been assembled from two different species of each genus (see Document S2). These sequences have been successfully used by previous authors to resolve relationships of caviomorph and/or cavioid rodents [13], [18], [26], [40] and were therefore thought to provide adequate levels of divergence for conducting molecular clock estimates.

#### Combined dataset

The phylogenetic dataset consisting of the 96 morphological characters and the 4014 characters of the four genes (Ghr, Tth, 12s, cyb) is available as Dataset S1 and also at DataDryad http://datadryad.org/ (doi:10.5061/dryad.v5p71).

#### Data analysis

The DNA sequences of each of the four genes (*12s*, *cyb*, *Ghr*, and *Tth*) were compiled from several previous analyses (see Document S2), and were aligned using CLUSTAL X [99] using the default values of gap opening (10/100) and gap extension (0.1). The leading and trailing gaps were replaced with missing entries. After alignment, the leading and trailing ends of the sequences with no homologous sequences in other species were deleted. The alignment resulted in 961 base pairs (bp) for *12s* gene, 1140 bp for *cyb* gene, 1099 bp for *Tth* gene, and 814 bp for *Ghr* gene.

The combined dataset of the 96 morphological characters was concatenated with the DNA sequences of the four genes (*12s*, *cyb*, *Ghr*, and *Tth*), scoring fossil taxa with missing entries for the DNA partitions. This dataset contained a total of 40 taxa and a total of 4110 characters. An equally weighted parsimony analysis was conducted treating gaps as missing data in TNT 1.1 [100], [101]. The heuristic search consisted in 1000 replicates of a Wagner tree with random addition sequence of taxa and followed by TBR branch swapping, collapsing zero-length branches under the strictest criterion. After this procedure, a final round of TBR branch swapping to find all most parsimonious trees (MPTs).

The results of the cladistic analysis (Fig. 1) are congruent with previous phylogenetic hypotheses [41], [47]–[49] and with the topology obtained in the Bayesian analysis of the molecular partition (see below). Further details on the parsimony analysis are given in the Document S1.

### Ghost Lineages and Calibrated Phylogenies

The timing and mode of evolutionary history of a group can be inferred by integrating the temporal information of the fossil record and the phylogenetic trees [102]. These two sources of temporal information are independent and can be compared to measure their congruence and to infer the existence of “ghost lineages” [103], [104], which extend the temporal range of a lineage prior to its appearance in the fossil record based on information from its sister lineage. Calibrating the phylogenetic trees that contain fossil taxa with the chronostratigraphic information (geologic time) provides minimal ages of divergence for each node of tree. We calibrated the phylogenetic trees using a script for TNT (see Script S1) and identify ghost lineages following the methodology proposed by previous authors, considering the age of first appearance of each terminal taxon in the fossil record as the only relevant temporal information [105].

### Molecular Clock Estimates

Bayesian analyses were conducted on the molecular data in BEAST v. 1.6 [106], performing a simultaneous estimation of the topology and divergence dates using a relaxed molecular clock. The four genes (*12s*, *cyb*, *Ghr*, and *Tth*) were input as separate partitions for the Bayesian analyses, model selection was performed using AIC (Akaike Information Criterion) [107] and hLRT (hierarchical Likelihood-Ratio Test) as implemented in Modeltest v. 3.1.2 [108]. The MCMC (Monte Carlo Markov Chain) was run using unlinked substitution models (GTR+Γ for all genes except for *cyb* that used GTR+I+Γ), a linked clock model (uncorrelated lognormal relaxed clock), and tree priors assuming a Yule process. Four independent MCMC runs of 10,000,000 generations (sampling every 1000 generations) were run independently for each of the 30 analyses we conducted using different calibration constraints (see below). Results of the four independent MCMC runs were integrated and summarized for checking convergence using the BEAST v. 1.6 package [106] and Tracer 1.5 [109]. The topologies sampled from the MCMC were summarized using TreeAnotator v. 1.6.2 [106] selecting the maximum clade credibility tree. This tree (Fig. 4) is identical to the one obtained using the majority rule consensus of the trees sampled in the MCMC runs and also recover the same relationships of extant lineages as we recovered from the parsimony analysis of the combined dataset (Figs. 1, 3), indicating the phylogenetic signal of the data is robust to the assumptions of the analysis (see Document S1).

#### Calibration constraints

Bayesian approaches to molecular clock estimates, as implemented in BEAST v. 1.6, allows using different prior probability distributions to calibrate selected node ages (calibration constraints). As noted by several authors in recent years [98], [110]–[115], the choice of calibration points is a critical step in molecular clock studies but is rarely discussed at length. We have explored the calibration of up to four nodes located both above and below the nodes of interest (diversification of caviid lineages). We performed 30 different analyses varying the number of calibrated nodes (from 1 to 4 calibrated nodes) and using two different prior distributions used for calibrating the age of these nodes (normal and gamma distributions; see below and Table 1). The analyses are numbered from 1 to 30, starting with the run with four constrained nodes that used a normal (analysis 1) or gamma (analysis 2) distribution. Analyses 3 to 10 used only three calibration constraints. Analyses 11 to 22 used two calibration constraints. Analyses 23 to 30 used a single calibration constraint.

Prior distributions of the ages of these four nodes were defined based on the available chronostratigraphic information of the fossil record, considering the phylogenetic placement of fossils in the phylogenetic analysis (see Fig. 1) and the uncertainties in the age of the fossiliferous sediments. Two different prior distributions were alternatively used (Normal and Gamma), which represent different levels of confidence on the true absence of a given lineage in sediments older than its first appearance in the fossil record.

Normal distributions were centered on midpoint age of the period of time to which the fossil-bearing formation has been referred. The standard deviation was set so that the 95% probability distribution reached the upper and lower bound of the age of the lithostratigrapic unit (see Calibrated Nodes and Table 1). This prior distribution makes a strong assumption on the absence of representatives of the calibrated node in older sediments, which may be appropriate in some cases but not in others [98].

Gamma distributions were used to produce asymmetric probability distributions that place the highest prior probability along the interval of time represented by the lithostratigrapic unit that has yielded the oldest member of the clade being calibrated. The long tail of the distribution extends towards older ages, gradually decreasing the probability (maximum soft-bound), and including in the 95% prior probability distribution the age of the most recent sediments in which representatives of the node being calibrated are absent, but numerous remains of its stem-group or other caviid lineages are known (see Calibrated Nodes and Table 1). The presence of abundant remains of outgroup taxa is a taphonomic-preservation control using ecological/taxonomic equivalents [116] and resembles the criteria advocated for calibrating nodes by some authors [98], [112], [114], [115], [117].

The 30 exploratory MCMC runs were conducted using different combinations of these distributions for the four calibration points and varying the number of nodes calibrated with the fossil record (from one to four calibrations; Table 1). The evidence used to define the prior distribution of ages of the four calibrated nodes is given in Calibrated Nodes and further details on the parameters used and the results obtained are given in Table 1.

### Calibrated Nodes

#### Node 1 (Cavioidea; see Figs.1, 4)

This node represents all cavioids and includes representatives of Dasyproctidae, Cuniculidae, and Cavioidea *sensu stricto*. The most ancient fossil that has been assigned to this clade is *Andemys termasi*, known from a mandibular fragment found in the Tinguiririca fauna from the Abanico Formation of Central Chile [24], [29], [118], dated at 31.5–37.5 Ma [61]. Its affinities have never been tested in a phylogenetic analysis, but the combination of plesiomorphic and apomorphic features identified in previous studies [24], [29]; mesodont crowns but with a deep hypoflexid that would have projected nearly to the lingual margin of the molars when unworn) indicates this specimen can be tentatively regarded as a basal member of Dasyproctidae and therefore useful in calibrating the age of the node Cavioidea.

Calibrating this node with *Andemys termasi* [29] from the Tinguiririca fauna involves two different sources of uncertainty. First, there is an uncertainty in the radiometric dates of the Abanico Formation. Wyss et al. [24] provided Ar/Ar dates of the fossiliferous horizons and concluded that the fossils are at least as old as 31.5 Ma but the deposition of lower levels of the unit must have started about 37.5 Ma. Therefore, the 31.5–37.5 Ma uncertainties should be taken into account when calibrating the age of this node. Second, as noted above, this fossil is the among the oldest cavioid rodent known from South America, being the earliest member of this large clade of caviomorph rodents that evolved after the arrival of ancestral hystricognaths to this continent. The timing of the arrival of rodents to South America is, however, poorly constrained given the scarce rodent fossil record prior to the Tinguirirican deposits. The recent discovery of the oldest rodent fauna in the Yahuarango Formation [27] lacks cavioid rodents and has been radioisotopically dated by Ar/Ar at 43.44±2.5 Ma, suggesting an upper bound for the origin of Cavioidea. This finding lies within the range of previous molecular clock estimates for the initial diversification of caviomorph rodents (after their arrival to South America) that ranged between 30.7 and 55 Ma [20], [56], [59]. Therefore, the actual time of diversification of Cavioidea is uncertain due to the poor record of Eocene caviomorphs and the error associated to radioisotopic dates of the oldest rodent faunas, as there is a minimum of 3.5 my and a maximum of 14.5 my between their appearance of cavioids in the fossil record (Tinguiririca) and the oldest rodent record from South America, in which cavioids are absent (Yahuarango).

We have explored the use of two different prior probability distributions to account for these sources of uncertainty while calibrating this node. The first approach uses a normal distribution whose 95% probability density encompasses the radioisotopic ages published for Tinguiririca (31.5–37.5 Ma). This distribution largely ignores the second source of uncertainty (i.e., lack of adequate early fossil record of Caviomorpha and Cavioidea) and places a strong belief in that the dasyproctid from Tinguiririca is actually very close (in time) to the divergence time of Cavioidea. The second approach uses a gamma distribution, with a hard minimum bound at 31.5 Ma and its long tail extends the 95% probability density back to 45 Ma, representing the upper bound of the available dates of oldest record of caviomorphs from South America [27], as well as the inferred date for the diversification of Caviomorpha obtained in the most densely sampled molecular clock analysis [59].

Finally, given the scarcity of the available material to constrain the age of this node and the lack of an explicit phylogenetic analysis including *Andemys termasi* [24], [29] Tinguiririca mandible, we have also tested the use of this fossil for calibrating the deepest node of our phylogenetic tree (Fig. 4). The results, however, are largely similar in terms of the molecular clock estimates for most nodes within Cavioidea and therefore we conducted all analyses using *Andemys termasi* to calibrate Cavioidea.

#### Node 2 (Cavioidea s.s. + Cuniculus paca; see Figs. 1, 4)

This node of the phylogenetic tree represents all forms of Cavioidea *s.s*. plus the lineage of the family Cuniculidae. The fossil record of Cuniculidae is extremely poor and only starts in the Pleistocene [119], [120]. Cavioidea *s.s*., in contrast, has an extremely rich fossil record [53], especially in the southern region of South America (Patagonia). The most ancient definitive members of Cavioidea *s.s.* are *Asteromys punctus* and *Chubutomys simpsoni,* both known from few specimens found in the late Oligocene beds of Patagonia (Deseadan SALMA) of the Sarmiento Formation [49], [121]. The phylogenetic position of both taxa within Cavioidea *s.s*. is strongly supported in by the morphological data of the phylogenetic analysis presented here (Fig. 1), as in previous phylogenetic studies [47]–[49]. In our analysis, the two most basal nodes of Cavioidea *s.s*. have high Bremer support (i.e., 4 and 5) and bootstrap and jackknife frequencies above 96% (Fig. 2; see Document S1).

The age of Deseadan deposits that yielded *Asteromys* and *Chubutomys* is therefore critical for calibrating this node. Specimens of *Asteromys* were found in the localities Cabeza Blanca and Laguna de los Machos [49] and the type *Chubutomys simpsoni* only in the former locality. These fossils were found together with a mammalian faunal assemblage characteristic of the Deseadan age [52]. No radiometric dates are available for these two localities but other Deseadan beds have been dated between 24.5 and 29 Ma [61]–[62]. The range of radiometric ages obtained for other Deseadan localities is the first source of uncertainty that should be taken into account for calibrating this node. The possibility that these early cavioid fossils are younger than the cladogenetic event that separated the cuniculid lineage from the lineage of Cavioidea *s.s*. is the second source of uncertainty that should be considered. Pre-Deseadan rodent assemblages from Patagonia are known from La Cantera locality, which lacks forms of Cavioidea *s.s*. ([65]; see below) and has been dated between 29.5 and 31.1 Ma [62].

As with the calibration of Node 1, we explored the use of two different prior probability distributions for this node. The first was a normal distribution whose 95% probability density encompasses the range of radioisotopic ages published for the Deseadan SALMA (24.5–29 Ma), ignoring the second source of uncertainty. The second approach uses a gamma distribution, which puts a hard minimum bound at 24.5 Ma and its long tail extends the 95% probability density back in time to the age of the youngest rodent assemblage that lacks forms of Cavioidea *s.s*. (i.e. 29.5–31.1Ma; [62]). Although we have explored both strategies we believe the second option more accurately represents the uncertainties of the early fossil record of cavioid rodents.

#### Node 3 (Kerodon + Hydrochoerus; see Figs. 1, 4)

This node of the molecular phylogeny represents all forms of Hydrochoerinae (including the lineage leading to the Rock cavy *Kerodon*). The fossil record of *Kerodon* is only known from scarce material of the late Pleistocene of Brazil [122]–[124]. Fossil representatives of the lineage leading to *Hydrochoerus* (see Fig. 1), in contrast, are relatively abundant in some Miocene-Pliocene deposits of South America [125]. The oldest definitive records of this lineage are found in the Arroyo Chasicó Formation of Central Argentina (Chasicoan SALMA) and include *Cardiatherium chasicoense* [126], [127], *Cardiomys cavinus* [128], and *Procardiomys martinoi* [129]. The phylogenetic position of *Cardiomys cavinus* and *Cardiatherium chasicoense* within Hydrochoerinae is supported by the morphological data of the phylogenetic analysis presented here (Fig. 1). Although the clades within Hydrochoerinae have low to moderate Bremer support values and bootstrap and jackknife frequencies are below 60% (Fig. 2; see Document S1), forcing the exclusion of both *Cardiomys* and *Cardiatherium* outside Hydrochoerinae requires five extra steps in the parsimony analysis, demonstrating their inclusion within Node 3 is relatively well supported by the morphological data.

The age of Chasicoan deposits yielding this diverse assemblage of hydrochoerines is important for calibrating this node. The stratigraphic levels that contain these taxa belong to the upper section of the Arroyo Chasicó Formation (Lithofacies 3 *sensu* Zárate [130] or Las Barrancas Member *sensu* Ugarte [131]. The age of these levels is certainly younger than 9.07 Ma, which is the age of radiometric dates of the underlying lithofacies 1 and 2 of the Arroyo Chasicó Formation [130]. Unfortunately, radiometric dates for the fossiliferous levels are lacking, but Deschamps [132] considered that lithofacies 3 of the Arroyo Chasicó Formation can be biostratigraphically correlated with the Loma de Las Tapias Formation (northwestern Argentina), which has a radiometric date of 7±0.9 Ma. A conservative approach, therefore, is to consider the age of the fossiliferous levels of the Arroyo Chasicó Formation as ranging between 6.1 and 9.07 Ma.

Although these fossils from the Chasicó Formation are the oldest well preserved and definitive record of hydrochoerines, it is difficult to determine if earlier members of this node were present before the deposition of this unit. In fact, the fossil record of cavioid rodents has a noticeable gap before the Chasicoan. The only older rodent remains that could be related to hydrochoerines come from the Mayoan SALMA (11.5 Ma; [133]) and consists of a single isolated tooth from the Río Frias Formation that has been referred with doubts to *Cardiomys* [80], [134]. This isolated tooth, however, lacks diagnostic features to determine their phylogenetic placement either as part of Node 3 or as a close relative of this clade. The caviid fossil record before the Mayoan is found in sediments of the Laventan SALMA (11.8–13.5Ma; [69]) and consists of the basalmost members Caviidae, which are definitively placed outside Hydrochoerinae (e.g., *Prodolichotis pridiana*; see below and Fig. 1), placing a maximal bound for the origin of this node.

As with the other nodes, we explored two different prior probability distributions for the age of Node 3. The first used a normal distribution whose 95% probability density encompasses the range of radioisotopic ages that bracket the fossiliferous levels of the Arroyo Chasicó Formation (6.1–9.07 Ma); this ignores the phylogenetic uncertainty of the fragmentary remains with possible hydrochoerine affinities in older sediments (e.g., Mayoan SALMA). The second used a gamma distribution, with a hard minimum bound at 6.1 Ma and a long tail to extend the 95% probability density back to the age of the Laventan SALMA, which is the youngest rodent assemblage that is well sampled and lacking forms that could potentially belong to Node 3 (i.e., 11.8–13.5 Ma). As in previous cases, we have explored both approaches but consider the second option better represents the uncertainties in the fossil record of early hydrochoerines.

#### Node 4 (*Microcavia* + *Cavia*; see Figs. 1, 4)

This node of the molecular tree represents all forms of Caviinae closer to *Microcavia* or *Cavia* than to *Galea*. The earliest fossil members of *Cavia* come from the San Andrés Formation (late Pliocene; [135]) and are based on scarce mandibular material. In contrast, fossils referred to *Microcavia* or other genera possibly related to *Microcavia* are relatively abundant in some Late Miocene to early Pliocene deposits of South America [124], [136]. The phylogenetic analysis performed here includes four fossil taxa that have been regarded as primitive members of Caviinae: *Allocavia chasicoense*, *Paleocavia impar*, *Dolicavia minuscula*, and *Microcavia chapalmalensis* [124], [134], [137], [138]. The last three taxa are retrieved as more closely related to the extant *Microcavia australis* than to *Cavia* and provide adequate information for calibrating this node.

*M. chapalmalensis* is recovered as the sister taxon of *M. australis* in the phylogenetic analysis (Fig. 1) and comes from the Chapadmalal Formation of Central Argentina (Chapadmalan SALMA). The sister group relationship of these two species of *Microcavia* are well supported by the morphological data of our phylogenetic analysis. The support values for the node of the genus *Microcavia* are low (Bremer  = 2 and bootstrap and jackknife frequencies around 60%; Fig. 2; see Document S1), but this is due to the unstable behavior in suboptimal (or bootstrap) trees of some fragmentary taxa (e.g., *Allocavia*). Forcing *M. chapalmalensis* to be positioned outside the Node 4 requires a minimum of five extra steps demonstrating the strong support for its inclusion within this node. The minimum age of the Chapadmalal Formation has been dated at 3.27 Ma using K-Ar radioisotopes [139], whereas the maximum age of these levels is usually regarded as 4 Ma [140], [141]. Fragmentary remains found in older sediments of Monte Hermoso Formation (Montehermosan SALMA) and Aconquija Formation (late Miocene – early Pliocene) have been referred to *Microcavia* [142], [143] but these cannot be identified at the species level and lack unambiguous synapomorphies of this genus. These remains could belong to the stem of *Microcavia* (i.e., being part of Node 4) or have an even more basal position, but more complete remains are needed to place them confidently.

*Dolicavia minuscula* was recovered in a basal polytomy within the lineage leading to the genus *Microcavia* together with *Paleocavia impar* (Fig. 1). Numerous and well-preserved remains of *Dolicavia* are known from the Chapadmalal Formation of Central Argentina ([134], [144]; 3.27–4 Ma). No remains of this taxon have been found in older deposits (e.g., Monte Hermoso Formation). In contrast to the strong support for the position of *Microcavia chapalmalensis*, the position of *Dolicavia* is only poorly supported, as it takes a single extra step to place this taxon basally within Caviinae and only 20% of the bootstrap and jackknife trees place this taxon within Node 4.

*Paleocavia impar* is known from multiple specimens with skull and mandibles found in the Monte Hermoso Formation [145]. As in the case of *Dolicavia*, the inclusion if *Paleocavia impar* within Node 4 is not strongly supported. Topologies with a single extra step places this taxon more basally within Caviinae and only 33% of the bootstrap and jackknife trees place *Paleocavia* allied with the genus *Microcavia*. The maximum age of this unit has been determined as 5.3 Ma based on radiometric dates [146] and the minimum age was estimated as 4 Ma [140], [141]. The presence of *Paleocavia* in older sediments (Huayquerian SALMA; 5.3–6.1 Ma; [147]) of the Ituzaingó Formation [148] and of the Cerro Azul Formation [149] have been reported in faunal lists of these units, but these specimens have not been described and we cannot test at the moment their phylogenetic affinities. If the presence of *Paleocavia* in the Ituzaingó and Cerro Azul formations were confirmed, it would push the first appearance of Node 4 back to the Huayquerian, but for the moment the Montehermosan record of *Paleocavia* is the oldest record of Node 4 (*Microcavia*+*Cavia*).

The rodent fossil record of the older sediments of the Chasicó Formation (Chasicoan SALMA; 6.1–9.07 Ma; see above) provides confident information to place a maximal bound for the origin of this node. The Chasicoan fossil record contains remains of caviine-like caviids (e.g., *Allocavia*) as well as numerous forms of Dolichotinae and Hydrochoerinae [53], [81], [150]. All these forms are recovered in the parsimony analysis outside the derived Node 4 of Caviinae (see Fig. 1). None of the hundreds of rodent remains known from Chasicoan deposits can be allied to the node formed by *Microcavia* and *Cavia* (Node 4).

As with other nodes, we explored two different prior probability distributions for the age of Node 4 based on the position of fossil taxa in the most parsimonious trees of our analysis. The first used a normal distribution whose 95% probability density encompasses the range of radioisotopic ages that bracket the fossiliferous levels of the Monte Hermoso Formation (4–5.3 Ma); this ignores the uncertainty associated with the possible presence of *Paleocavia* in older sediments (i.e., Huayquerian). The second approach used a gamma distribution, with a hard minimum bound at 4 Ma and a long tail that extended the 95% probability density back to the Chasicoan, which is the youngest well-known rodent assemblage lacking taxa that belong to Node 4 (i.e., 6.1–9.07 Ma). As in previous cases, we have explored both approaches but believe the second option better represents the uncertainties in the fossil record of early caviines.

Finally, given the oldest member of this clade (*Paleocavia*; Montehermosan SALMA) is only poorly supported within Node 4, we have also tested alternative calibrations for this node. We have conducted exploratory runs of the Bayesian analysis calibrating this node with the age of *Microcavia chapalmalensis* (Chapadmalan SALMA), which is the only fossil of this clade that is robustly supported by the morphological data of the phylogenetic analysis (see above). The results of this analysis are largely similar in terms of the molecular clock estimates for Caviidae (mean age  = 17.5 Ma) and place the diversification of dolichotines and *Galea* within the Chasicoan SALMA. Therefore, the estimates of interest for our purposes do not seem to be sensitive to the alternative ages that can potentially be used for calibrating Node 4.

## Supporting Information

Document S1 **Supporting information of the phylogenetic analysis conducted on the combined dataset.**
(DOC)

Document S2 **List of taxa used for the Phylogenetic Analysis and GenBank accession numbers.**
(DOC)

Document S3 **List of morphological characters used in the phylogenetic analysis.**
(DOC)

Dataset S1 **Combined data matrix containing molecular and morphological characters in Nexus format.**
(TNT)

Script S1 Script for calibrating phylogenetic trees using the chronostratigraphic information for fossil taxa in TNT (calculates MSM*, GER, and provides a calibrated topology in nexus format).
(TXT)
