# Supplementary material for: Major Radiations in the Evolution of Caviid Rodents: Reconciling Fossils, Ghost Lineages, and Relaxed Molecular Clocks
Source: PLoS One. 2012 Oct 29;7(10):e48380. doi: 10.1371/journal.pone.0048380 (PMC3483234; doi:10.1371/journal.pone.0048380)
Supplement: Document S1 — Supporting information of the phylogenetic analysis conducted on the combined dataset. (DOC) [file pone.0048380.s001.doc]

**Document S1** – **Phylogenetic Analysis**

Combined Parsimony Analysis (morphology+DNA)

The initial heuristic search found MPTs in 734 out of the 1000 replicates. The second round of TBR branch swapping resulted in a total of 192 trees of 3164 steps (CI=0.645 RI=0.476). The results of the cladistic analysis yielded the same topological relationships of the extant taxa than the topology obtained in the Bayesian analysis of the molecular partition.

The 192 MPTs differ in the interrelationship of basal euhypsodont cavioids (species of the genus *Eocardia*, *Schistomys*, and *Matiamys*) and in the alternative positions of the fragmentary taxon *Allocavia*. The alternative positions of *Allocavia* collapse several basal nodes of the crown group Caviidae in the strict consensus tree:

**
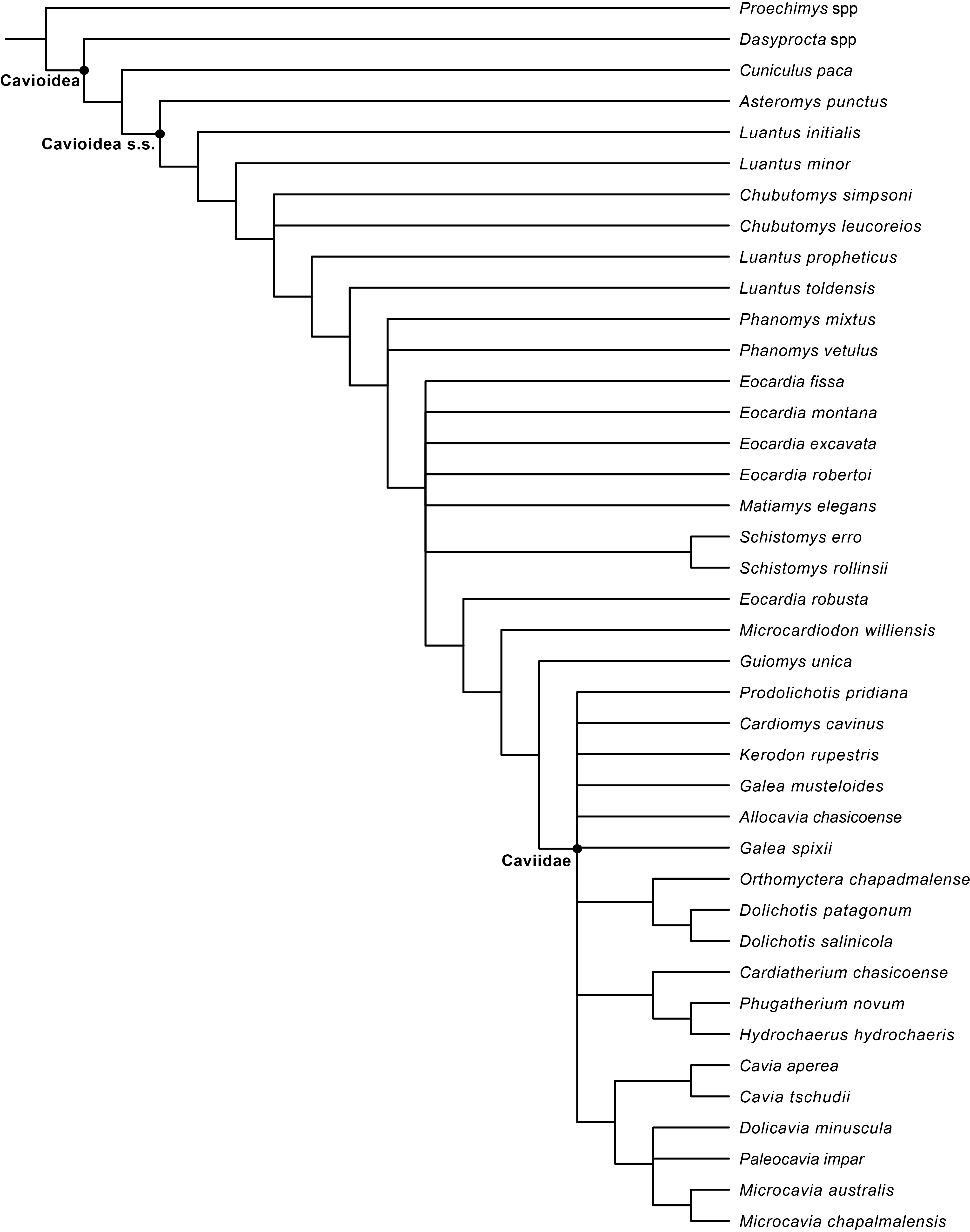
**

**Figure S1.1**. Strict consensus tree including all taxa of the combined phylogenetic analysis.

When the alternative positions of the fragmentary fossil taxon *Allocavia* are ignored the reduced consensus tree reveals that the 192 MPTs have a common topology that resolves the interrelationships of the three major lineages of Caviidae: Caviinae, Dolichotinae, and Hydrochoerinae. This is shown by the reduced consensus (Fig. S1.2).

*
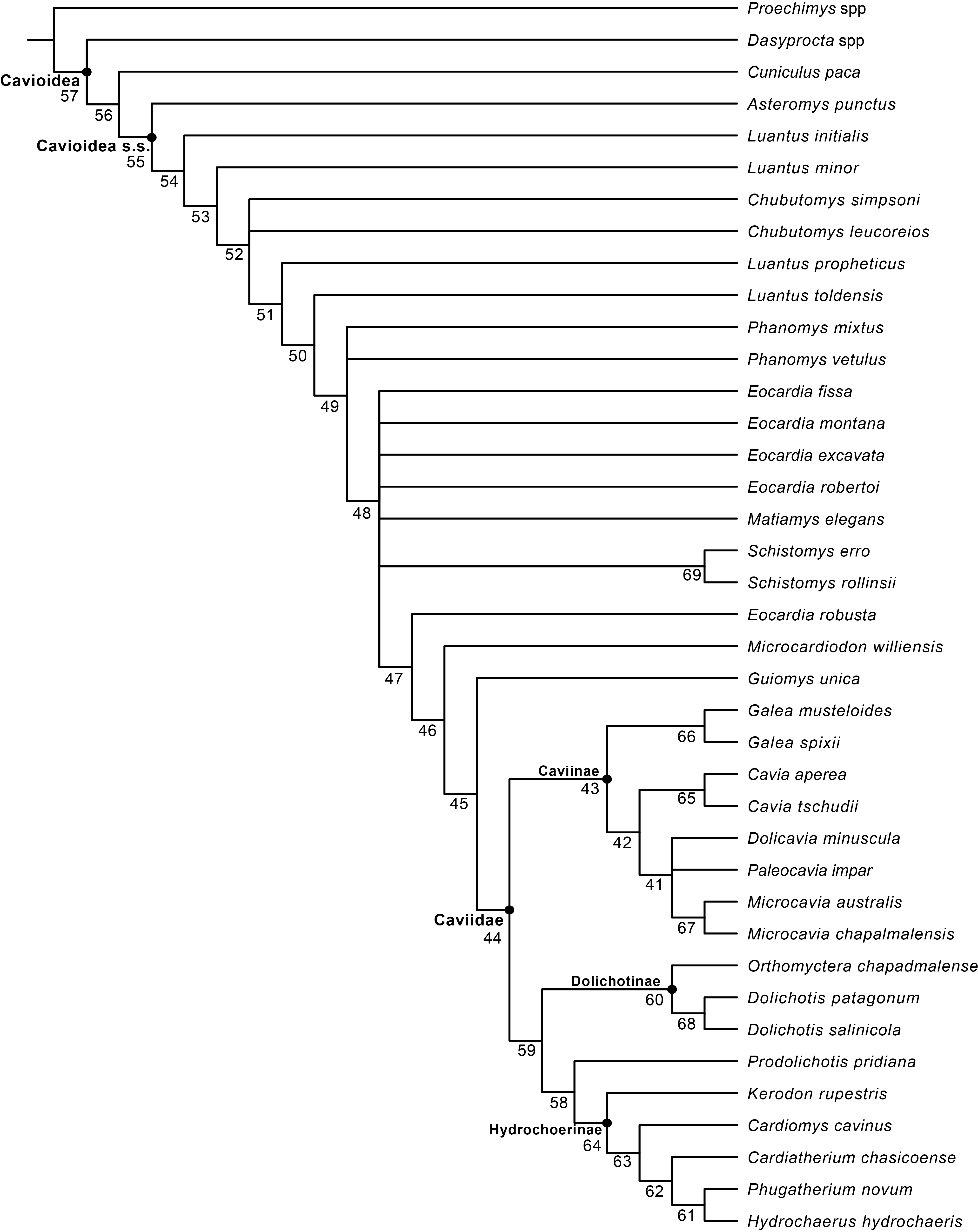
*

**Figure S1.2**. Reduced strict consensus tree pruning *Allocavia* from the 192 MPTs obtained in the combined phylogenetic analysis.

List of Unambiguous Synapomorphies

The following list details the unambiguous synapomorphies found in the parsimony analysis of the combined analysis (ignoring the position of the unstable and fragmentary fossil taxon *Allocavia*). The listed characters are unambiguous synapomorphies found in the 192 MPTs, except those that are optimized only in some of them that are indicated as found “In some trees”. Node numbers refer to those given in Figure S1.2.

Node 41:

All trees:

Char. 17: 2 --> 3

Char. 62: 1 --> 0

Char. 80: 1 --> 0

Some trees:

Char. 5: 0 --> 1

Node 42:

All trees:

Char. 13: 1 --> 2

Char. 23: 1 --> 0

Char. 25: 2 --> 1

Char. 113: A --> G

Char. 115: T --> C

Char. 135: G --> A

Char. 142: C --> T

Char. 166: A --> T

Char. 176: A --> T

Char. 195: T --> C

Char. 228: A --> G

Char. 255: A --> G

Char. 308: A --> G

Char. 380: T --> C

Char. 427: T --> C

Char. 460: A --> G

Char. 492: A --> G

Char. 570: C --> T

Char. 757: A --> G

Char. 898: T --> C

Char. 914: A --> G

Char. 972: A --> G

Char. 1108: T --> C

Char. 1161: AT --> C

Char. 1338: T --> A

Char. 1353: A --> G

Char. 1503: C --> T

Char. 1569: C --> T

Char. 1725: C --> T

Char. 1744: A --> T

Char. 2276: T --> C

Char. 2352: C --> T

Char. 2758: T --> C

Char. 2884: G --> A

Char. 2965: A --> C

Char. 3612: A --> C

Char. 3804: A --> C

Char. 3820: T --> C

Char. 3948: A --> C

Char. 4010: T --> C

Node 43:

All trees:

Char. 12: 2 --> 0

Char. 18: 2 --> 1

Char. 95: 1 --> 0

Char. 192: A --> T

Char. 333: T --> C

Char. 564: T --> C

Char. 694: T --> C

Char. 801: A --> G

Char. 981: A --> T

Char. 1086: A --> C

Char. 1476: C --> T

Char. 1710: C --> T

Char. 2185: C --> T

Char. 2295: T --> C

Char. 2329: T --> C

Char. 2353: A --> G

Char. 2736: C --> T

Char. 3591: T --> C

Char. 3755: G --> A

Char. 3788: A --> G

Char. 4088: G --> A

Some trees:

Char. 88: 0 --> 1

Node 44:

All trees:

Char. 2: 0 --> 1

Char. 19: 1 --> 2

Node 45:

All trees:

Char. 19: 0 --> 1

Char. 20: 1 --> 2

Char. 22: 1 --> 2

Char. 24: 2 --> 3

Char. 35: 0 --> 1

Char. 36: 0 --> 1

Char. 44: 1 --> 2

Node 46:

All trees:

Char. 50: 0 --> 1

Node 47:

All trees:

Char. 32: 2 --> 3

Some trees:

Char. 93: 1 --> 2

Node 48:

All trees:

Char. 28: 2 --> 3

Char. 47: 0 --> 1

Some trees:

Char. 45: 5 --> 4

Char. 49: 0 --> 1

Node 49:

All trees:

Char. 29: 1 --> 2

Char. 32: 1 --> 2

Some trees:

Char. 51: 0 --> 1

Node 50:

All trees:

Char. 43: 0 --> 1

Char. 46: 0 --> 1

Node 51:

All trees:

Char. 24: 1 --> 2

Char. 42: 0 --> 1

Node 52:

All trees:

Char. 45: 0 --> 2

Node 53:

All trees:

Char. 28: 1 --> 2

Node 54:

All trees:

Char. 30: 0 --> 1

Char. 31: 0 --> 1

Char. 36: 1 --> 0

Char. 41: 0 --> 1

Char. 44: 0 --> 1

Node 57:

All trees:

Char. 3: 0 --> 1

Char. 21: 0 --> 1

Char. 29: 0 --> 1

Char. 32: 0 --> 1

Node 58:

All trees:

Char. 9: 0 --> 1

Char. 15: 0 --> 1

Char. 20: 0 --> 1

Char. 23: 0 --> 1

Char. 24: 0 --> 1

Char. 25: 0 --> 1

Char. 60: 1 --> 0

Char. 391: G --> A

Char. 449: A --> T

Char. 469: G --> A

Char. 515: T --> C

Char. 570: T --> C

Char. 709: T --> C

Char. 766: A --> T

Char. 856: C --> T

Char. 871: G --> A

Char. 876: T --> C

Char. 904: C --> T

Char. 1107: T --> A

Char. 1116: T --> C

Char. 1123: A --> G

Char. 1254: T --> C

Char. 1257: A --> C

Char. 1380: T --> A

Char. 1414: T --> C

Char. 2207: C --> A

Char. 2223: C --> T

Char. 2517: A --> G

Char. 2546: G --> T

Char. 2730: T --> C

Char. 2748: A --> G

Char. 2793: T --> G

Char. 4047: G --> A

Node 57:

All trees:

No synapomorphies

Node 58:

All trees:

Char. 44: 2 --> 3

Char. 58: 1 --> 0

Node 59:

All trees:

Char. 4: 1 --> 0

Char. 76: 1 --> 0

Char. 100: C --> T

Char. 316: A --> T

Char. 404: A --> G

Char. 967: T --> C

Char. 1098: T --> C

Char. 1179: A --> C

Char. 1182: C --> A

Char. 1320: A --> C

Char. 1419: C --> T

Char. 1749: G --> A

Char. 1804: T --> C

Char. 1806: A --> CT

Char. 1809: A --> T

Char. 1818: T --> C

Char. 1851: A --> C

Char. 1938: A --> C

Char. 2014: A --> T

Char. 2593: C --> A

Char. 2737: T --> C

Char. 3021: A --> T

Char. 3740: G --> A

Node 60:

All trees:

Char. 11: 1 --> 2

Char. 27: 0 --> 1

Char. 64: 1 --> 0

Char. 75: 0 --> 1

Char. 77: 1 --> 0

Char. 94: 0 --> 1

Node 61:

All trees:

Char. 56: 1 --> 2

Char. 88: 1 --> 2

Char. 89: 4 --> 5

Node 62:

All trees:

Char. 32: 3 --> 4

Char. 89: 3 --> 4

Node 63:

All trees:

Char. 59: 1 --> 0

Char. 89: 2 --> 3

Char. 93: 2 --> 3

Node 64:

All trees:

Char. 13: 01 --> 2

Char. 17: 2 --> 3

Char. 18: 2 --> 1

Char. 44: 3 --> 4

Char. 79: 1 --> 0

Char. 88: 0 --> 1

Node 65:

All trees:

Char. 91: 0 --> 1

Char. 93: 2 --> 1

Node 66:

All trees:

Char. 17: 2 --> 1

Char. 118: C --> T

Char. 122: A --> C

Char. 158: G --> A

Char. 305: G --> A

Char. 368: A --> T

Char. 408: T --> C

Char. 418: A --> T

Char. 421: A --> G

Char. 438: T --> C

Char. 447: A --> G

Char. 450: C --> T

Char. 483: T --> C

Char. 551: A --> G

Char. 558: T --> A

Char. 687: C --> T

Char. 758: C --> T

Char. 766: T --> A

Char. 807: G --> A

Char. 897: T --> C

Char. 915: A --> G

Char. 973: A --> T

Char. 983: C --> A

Char. 1080: C --> T

Char. 1123: G --> A

Char. 1131: C --> A

Char. 1177: C --> T

Char. 1212: A --> T

Char. 1234: A --> C

Char. 1235: C --> T

Char. 1251: T --> A

Char. 1296: A --> C

Char. 1342: C --> A

Char. 1422: A --> C

Char. 1434: CT --> A

Char. 1533: C --> A

Char. 1534: C --> T

Char. 1575: C --> A

Char. 1624: G --> T

Char. 1734: C --> T

Char. 1753: C --> A

Char. 1794: A --> C

Char. 1824: C --> T

Char. 1839: A --> T

Char. 1866: A --> T

Char. 1875: C --> T

Char. 1902: C --> A

Char. 1908: C --> T

Char. 1914: C --> T

Char. 1944: C --> A

Char. 1956: C --> T

Char. 1986: A --> C

Char. 2102: C --> T

Char. 2106: C --> T

Char. 2148: C --> T

Char. 2168: T --> C

Char. 2172: A --> C

Char. 2221: A --> G

Char. 2236: T --> C

Char. 2240: G --> A

Char. 2284: T --> G

Char. 2341: A --> G

Char. 2347: A --> C

Char. 2348: T --> C

Char. 2402: C --> T

Char. 2403: A --> G

Char. 2575: G --> A

Char. 2592: C --> T

Char. 2593: C --> T

Char. 2629: T --> C

Char. 2650: T --> A

Char. 2693: T --> G

Char. 2722: T --> G

Char. 2987: C --> G

Char. 2993: A --> T

Char. 3011: T --> C

Char. 3042: A --> G

Char. 3104: G --> A

Char. 3134: T --> C

Char. 3136: T --> G

Char. 3162: T --> A

Char. 3230: T --> G

Char. 3270: T --> C

Char. 3349: A --> G

Char. 3477: G --> A

Char. 3594: C --> T

Char. 3638: A --> T

Char. 3651: C --> T

Char. 3655: T --> A

Char. 3706: C --> T

Char. 3825: C --> T

Char. 3888: G --> A

Char. 4011: C --> G

Char. 4052: T --> C

Char. 4071: G --> A

Char. 4085: C --> T

Some trees:

Char. 14: 0 --> 1

Node 67:

All trees:

Char. 18: 1 --> 2

Char. 35: 1 --> 0

Char. 36: 1 --> 0

Char. 63: 0 --> 1

Node 68:

All trees:

Char. 15: 1 --> 2

Char. 59: 1 --> 0

Char. 82: 1 --> 0

Node 69:

All trees:

Char. 50: 0 --> 1

Some trees:

Char. 93: 1 --> 2

Nodal Support

The nodal support of the combined analysis was evaluated using Bremer support [4], bootstrap [5], and jackknife [6]. As in the case of the list of synapomorphies the nodal support values was calculated reduced consensus trees pruning *Allocavia* after the analysis, both for the Bremer analysis and for the resampling methods (bootstrap and jackknife). It should be noted that the fragmentary taxon *Allocavia* was included in the analysis of the suboptimal topologies (Bremer support) and in the heuristic tree searches performed for each of the bootstrap/jackknife replicates, and it was only pruned after this analysis was completed.

Bremer support values were calculated using the TNT script BREMER.RUN included in the TNT software package [2,3], which performs a combined search of suboptimal trees and negative constraints to search the most parsimonious trees that lack each node as monophyletic.

For both resampling techniques (bootstrap and jackknife) we performed 1000 replicates and a heuristic tree search. In addition to the absolute frequency of each node in each of the 1000 replicates we also calculated the GC frequency (as described by [7]). The GC frequency of each node is obtained by substracting the frequency of the most common clade that contradicts a given node to the absolute frequency of that particular node in the 1000 replicates of bootstrap or jackknife.


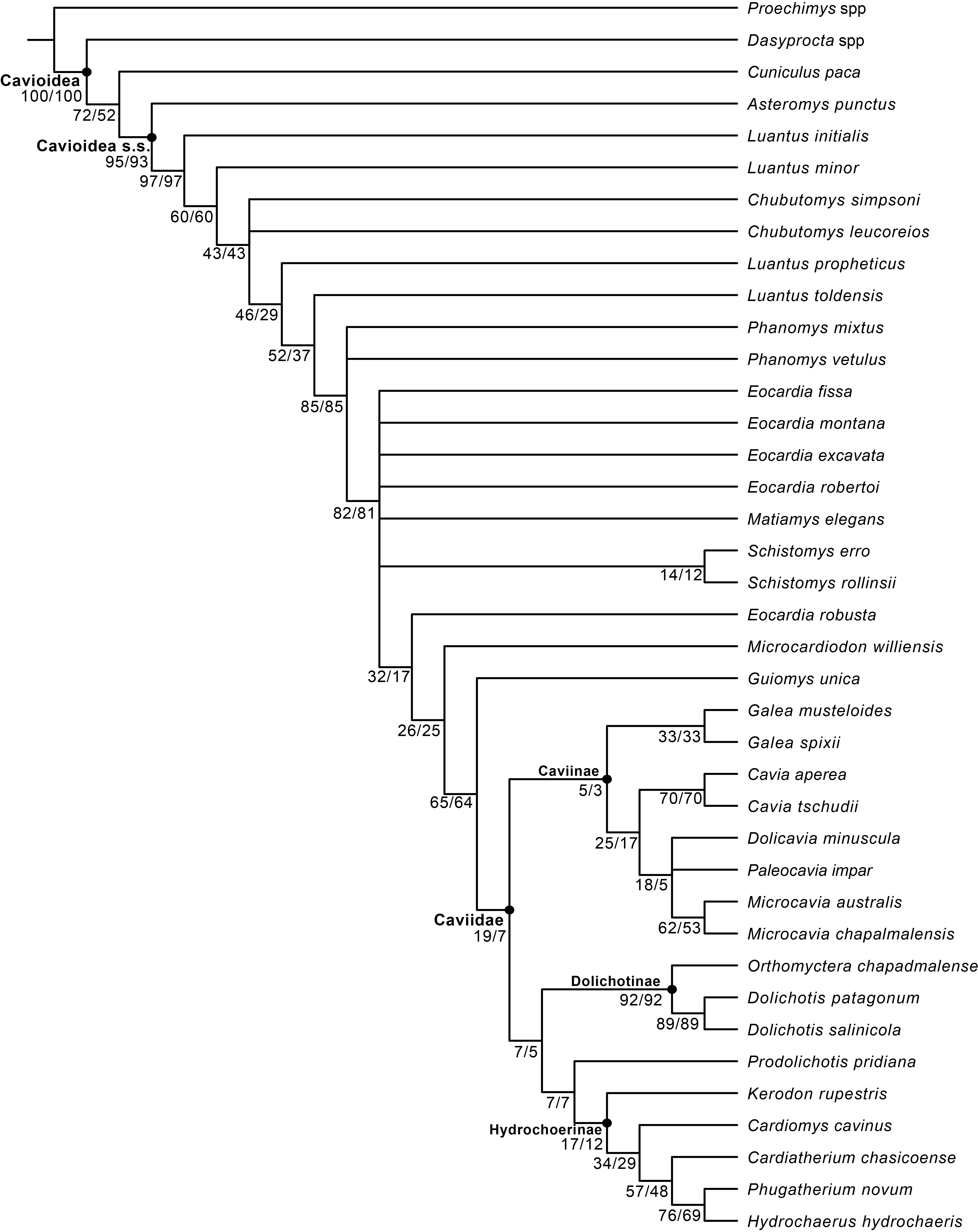


**Figure S1.3**. Bootstrap support values. Two values are indicated for each node, the first of them is the absolute frequency of each node in the bootstrap replicates and the second value is the GC frequency of each node.


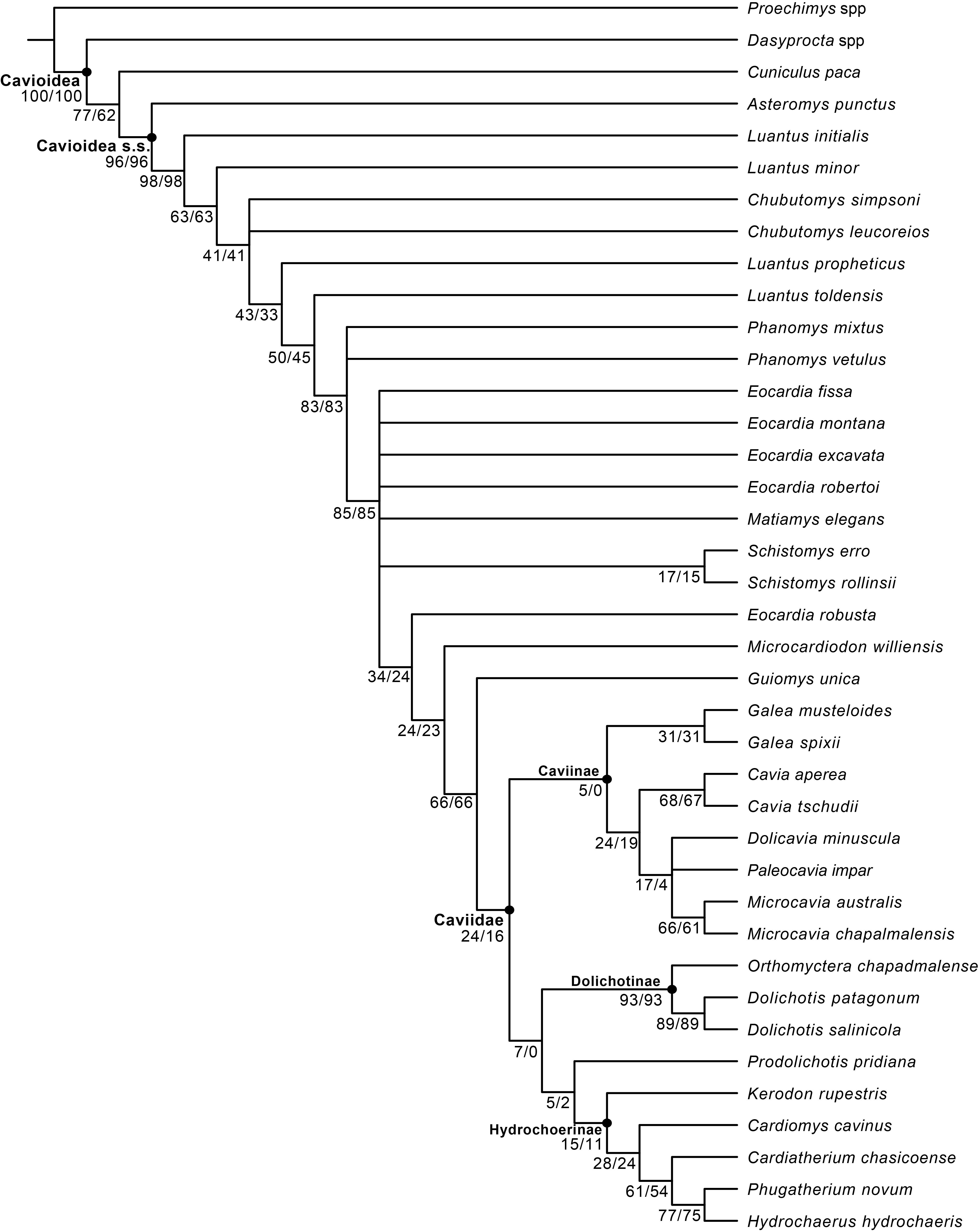


**Figure S1.4**. Jackknife support values. Two values are indicated for each node, the first of them is the absolute frequency of each node in the jackknife replicates and the second value is the GC frequency of each node.

**References**

1. Thompson JD, [Gibson TJ,](http://nar.oxfordjournals.org/search?author1=Toby+J.+Gibson&sortspec=date&submit=Submit) [Plewniak](http://nar.oxfordjournals.org/search?author1=Frédéric+Plewniak&sortspec=date&submit=Submit) F, [Jeanmougin](http://nar.oxfordjournals.org/search?author1=François+Jeanmougin&sortspec=date&submit=Submit) F, Higgins DG (1997) The CLUSTAL_X windows interface: flexible strategies for multiple sequence alignment aided by quality analysis tools. Nucleic Acids Res 25:4876-4882.
2. Goloboff P, Farris J, Nixon K (2008) TNT: Tree analysis using new technology, version 1.1 (Willi Hennig Society Edition). [<http://www.zmuc.dk/public/phylogeny/tnt>]
3. Goloboff P, Farris J, Nixon K (2008) A free program for phylogenetic analysis. Cladistics 24:774-776.
4. Bremer K (1994) Branch support and tree stability. Cladistics 10:295-304.
5. Felsenstein J (1985) Confidence limits on phylogenies: an approach using the bootstrap. Evolution 39:783-791.
6. Farris J, Albert V, Källersjö M, Lipscomb D, Kluge A (1996) Parsimony jackknifing outperforms neighbor-joining. Cladistics 12:99-124.
7. Goloboff P, Farris J, Kallersjo M, Oxelman B, Ramirez M (2003) Improvements to resampling measures of group support. Cladistics 19:324-332.
