# Supplementary material for: Major Radiations in the Evolution of Caviid Rodents: Reconciling Fossils, Ghost Lineages, and Relaxed Molecular Clocks
Source: PLoS One. 2012 Oct 29;7(10):e48380. doi: 10.1371/journal.pone.0048380 (PMC3483234; doi:10.1371/journal.pone.0048380)
Supplement: Document S2 — List of taxa used for the Phylogenetic Analysis and GenBank accession numbers. (DOC) [file pone.0048380.s002.doc]

**Document S2 – Taxa used for the Phylogenetic Analysis († = extinct)**

| Family | Species |
| --- | --- |
| Caviidae | †*Allocavia chasicoense* |
|  | *Cavia aperea* |
|  | *Cavia tschudii* |
|  | †*Cardiatherium chasicoense* |
|  | †*Cardiomys cavinus** |
|  | †*Dolicavia minuscula* |
|  | *Dolichotis patagonum* |
|  | *Dolichotis salinicola* |
|  | *Galea musteloides* |
|  | *Galea spixii* |
|  | *Hydrochoerus hydrochaeris* |
|  | *Kerodon rupestris* |
|  | *Microcavia australis* |
|  | †*Microcavia chapadmalensis* |
|  | †*Orthomyctera chapadmalense* |
|  | †*Paleocavia impar* Ameghino |
|  | †*Phugatherium novum* Ameghino |
|  | †*Prodolichotis pridiana* |
| “Eocardiidae” | †*Asteromys punctus* Ameghino |
|  | †*Chubutomys leucoreios* |
|  | †*Chubutomys simpsoni* |
|  | †*Eocardia excavata* |
|  | †*Eocardia fissa* |
|  | †*Eocardia montana* |
|  | †*Eocardia robertoi* |
|  | †*Eocardia robusta* |
|  | †*Guiomys unica* |
|  | †*Luantus initialis* |
|  | †*Luantus minor* |
|  | †*Luantus propheticus* |
|  | †*Luantus toldensis* |
|  | †*Matiamys elegans* |
|  | †*Microcardiodon williensis* |
|  | †*Phanomys mixtus* Ameghino |
|  | †*Phanomys vetulus* Ameghino |
|  | †*Schistomys erro* Ameghino |
|  | †*Schistomys rollinsii* |
| Cuniculidae | *Cuniculus paca* |
| Dasyproctidae | *Dasyprocta azarae* |
| Echimyidae | *Proechimys poliopus* |

†*Cardiomys cavinus** was based on the specimens MLP 55-IV-28-11, MMP 1507-M, and MMP 278-M. Acronyms: MLP, Museo de La Plata, Argentina; MMP, Museo de Mar del Plata, Argentina.

**GenBank accession numbers for exemplar taxa used in molecular analysis.**

^*^ The presence of that taxon in the morphological matrix.

| **Family** | **Species** | ***Tth*** | ***Ghr*** | ***cyb*** | ***12s*** |
| --- | --- | --- | --- | --- | --- |
| Caviidae | ^*^*Cavia aperea* | AF433883.1 | AF433930.1 | GU136759.1 | AF433908.1 |
|  | ^*^*Dolichotis patagonum* | AF433893.1 | AF433939.1 | AY382787.1 | AF433917.1 |
|  | ^*^*Dolichotis salinicola* | AF433895.1 | AF433941.1 | GU136723.1 | AF433919.1 |
|  | ^*^*Galea musteloides* | AF433885.1 | AF433932.1 | GU067527.1 | AF433910.1 |
|  | ^*^*Galea spixii* | AF433888.1 | AF433935.1 | GU067492.1 | AF433913.1 |
|  | ^*^*Hydrochoerus hydrochaeris* | AF433902.1 | AF433948.1 | GU136721. | U12454.1 |
|  | ^*^*Kerodon rupestris* | AF433891.1 | AF433938.1 | GU136722.1 | AF433916.1 |
|  | ^*^*Microcavia australis* | AF433889.1 | AF433937.1 | AF491750.1 | AF433915.1 |
| Cuniculidae | ^*^*Cuniculus paca* | AF433880.1 | AF433928.1 | AY206570.1 | AF520693.1 |
| Dasyproctidae | *Dasyprocta leporina* | not available | not available | AF437783.1 | not available |
|  | *Dasyprocta punctata* | AF433897.1 | AF433942.1 | not available | AF433921.1 |
| Echimyidae | *Proechimys longicaudatus* | FJ865463.1 | AF332039.1 | not available | U12447.1 |
|  | *Proechimys simonsi* | not available | not available | U35414.1 | not available |
