# Supplementary material for: Major Radiations in the Evolution of Caviid Rodents: Reconciling Fossils, Ghost Lineages, and Relaxed Molecular Clocks
Source: PLoS One. 2012 Oct 29;7(10):e48380. doi: 10.1371/journal.pone.0048380 (PMC3483234; doi:10.1371/journal.pone.0048380)
Supplement: Document S3 — List of morphological characters used in the phylogenetic analysis. (DOC) [file pone.0048380.s003.doc]

**Document S3 – Morphological Character List**

Characters 1 through 89 are based on the dataset of Pérez and Vucetich [1,2] and characters 90 through 96 have been added for this study.

(1) Length of the upper diastema: equal or longer then the molariform series (0); shorter than molariform series (1).

(2) Mental foramen: absent (0); present (1).

(3) Location of the mental foramen on the anterior region of the dentary: close to the dorsal margin of the dentary and opening dorsolaterally (0); at the dorsoventral midpoint of the lateral surface of the dentary and opening laterally (1).

(4) Position of the mandibular foramen: behind the retromolar fossa (0); below the m3 (1).

(5) Posteroventral projection of the posterior end of the mandibular symphysis: absent (0); present (1).

(6) Development of posteroventral projection of the posterior end of the mandibular symphysis in lateral view: well developed, forming an elongate peg exposed in lateral view (0); moderately developed, only a low bulge projects ventrally and is marginally exposed in lateral view (1).

(7) Labial edge of the condyle that is the insertion point of *m. masseter posterior*, in posterior view: projecting laterally with respect to wall of the dentary, forming small knob (0); lacking a distinct knob, continuous with lateral wall of the dentary (1).

(8) Medial edge of the condyle that is the insertion point of *m. pterygoideus externus*, in posterior view: projecting medially forming a shelf that overhangs the medial surface of the dentary (0); poorly developed projecting medially forming a small knob with respect to medial wall of the dentary (1).

(Continued)

(9) Shape of the post-condylar process, in lateral view: squared-off, forming approximately a 90° angle (0); rounded (1).

(10) Length of the post-condylar process: equal or longer than the anteroposterior length of the condyle (0); shorter than anteroposterior length of the condyle (1).

(11) Height of the coronoid process compared to the position of the condyle: located at the same dorsoventral level as the condyle (0); located more ventrally than the condyle (1).

(12) Anterior margin of the coronoid process: convex (0); straight (1); concave (2). This character treated as ordered.

(13) Dorsal end of the coronoid process: pointed and posterodorsally projected (0); pointed and dorsally projected (1); blunt (2).

(14) Dorsoventral position of the mandibular notch: located above the occlusal surface of the dental series (0); located at the same height as the occlusal surface of the dental series (1); located ventral to the occlusal surface of the dental series (2). This character treated as ordered.

(15) Shape of the mandibular notch: concave (0); almost straight (1).

(16) Dorsoventral position of the anterior most point of the lunar notch: low, located ventral to the dorsoventral midpoint of the dentary (between the ventral edge of the dentary and the condyle) (0); located at the approximate dorsoventral midpoint of the dentary (1); high, located above the dorsoventral midpoint of the dentary (2). This character treated as ordered.

(Continued)

(17) Posterior extension of the angular process: level with the post-condylar process (0); ending anterior to the post-condylar process (1); ending posterior to the post-condylar process (2).

(18) Posterior extension of the root of the lower incisors: extending up to the level of m3 (0); extending up to the level of the posterior lobe of m2 (1); extending up to the level of the anterior lobe of m2 (2); extending up to the level of m1 (3). This character treated as ordered.

(19) Location of the notch for the insertion of the tendon of the *m. masseter medialis pars infraorbitalis* with respect to the toothrow: between p4 and m1 (0); below m1 (1); between m1 and m2 (2). This character treated as ordered.

(20) Notch for the insertion of the tendon of the *m. masseter medialis pars infraorbitalis*: connected to the masseteric crest (0); isolated, located between the massteric crest and the horizontal crest (1); connected to the horizontal crest (2). This character treated as ordered.

(21) Development of the masseteric crest: well developed, forming a shelf that projects laterally with respect to the lateral surface of the dentary (0); forming a well-developed ridge that fails to project with respect to the lateral surface of the dentary (1); absent or poorly developed as a thin and low ridge (2). This character treated as ordered.

(22) Shape of the lateral crest (*sensu* Woods [3]): straight, projecting anteroventrally from the base of the coronoid process (0); curved, deflecting anteroventrally from the base of the coronoid process (1).

(23) Anterior origin of the masseteric crest with respect to the toothrow: below m1 (0); below m2 (1); below m3 or posteriorly to m3 (2). This character treated as ordered.

(Continued)

(24) Posterior extension of the horizontal crest, in lateral view: extending up to the anterior margin of the mandibular condyle (0); approximately ending at the anteroposterior midpoint of the mandibular condyle (1); extending up to the posterior margin of the mandibular condyle (2). This character treated as ordered.

(25) Development of the horizontal crest: absent or extremely reduced (0); present as a low and broad ridge (1); present as a conspicuous crest, forming a laterally projected shelf but lacking a dorsal fossa (2); well developed, forming a laterally projected shelf and bearing a fossa on its dorsal surface (3). This character treated as ordered.

(26) Depth of the fossa located dorsal to the horizontal crest with respect to the dorsoventral depth of the notch for the insertion of the tendon of the *m. masseter medialis pars infraorbitalis*: notch deeper than fossa (0); fossa deeper than notch (1); notch and fossa equal in depth (2).

(27) Alveolar protuberances (ventral outgrowth of the base of some molariform alveoli that projects ventrally from the ventral surface of the dentary): absent (0); present (1).

(28) Development of alveolar protuberances: present as a small but distinct convexity on the ventral margin of the dentary (0); present as well-developed bulge on the ventral margin of the dentary (1).

(29) Degree of hypsodonty: slightly hypsodont, having the root and the anteroposterior length of the occlusal surface longer than the height of the crown (0); mesodont, having the root and the anteroposterior length of the occlusal surface approximately equal to the height of the crown (1); protohypsodont, having the root and the anteroposterior length of the occlusal surface less than half the height of the crown (2); euhypsodont, lacking roots (3). This character treated as ordered.

(Continued)

(30) Shape of the m1-m2, in oclussal view: not forming lobes (0); formed by triangular lobes (1); formed by heart-shaped lobes (2); formed by two laminar lobes, the posterior one being formed by a single lamina (3).

(31) Constriction of the apex in each lobe of the molars: absent (0); present (1).

(32) Transverse extension of the hypoflexus/id: transversely shorter than half of the width of the crown (0); extending from the margin up to the transverse midpoint of the crown (1); extending beyond the transverse midpoint of the crown (2). This character treated as ordered.

(33) Shape of the hypoflexus/id in occlusal view: very narrow and short (0); V-shaped (1); narrow and very long (2); funnel shaped (3); canal shaped (4).

(34) Longitudinal furrow on the labial wall of the upper molars and lingual wall of the lower molars: absent (0); present (1).

(35) Transverse dentine crest on the occlusal surface, located at the middle of each molar lobe: absent (0); present (1).

(36) Length of p4-m1 with respect to the length of the m2-m3 [4]: p4-m1 shorter than m2-m3 (0); p4-m1 approximately equal to m2-m3 (1).

(37) Relative size of lower molars: m1 < m2<m3 (0); m1=m2=m3 (1).

(38) Relative size of the upper molars: P4 < M1 < M2 (0); P4=M1=M2 (1).

(39) Replacement of deciduous premolar: unreplaced (0); with replacement (1).

(40) Type of replacement: postnatal replacement (0); prenatal replacement (1).

(41) Orientation of left and right molar series: parallel to each other (0); anteriorly convergent (1).

(42) Cement in late ontogenetic stages: absent (0); present (1).

(Continued)

(43) Cement in young-adult ontogenetic stages: absent (0); present (1).

(44) Cement in juvenile ontogenetic stages: absent (0); present (1).

(45) Lobes in p4: two incipient lobes (0); two well-developed lobes, but lacking an anterior projection (1); two lobes and one incipient anterior projection that is not separated from the anterior lobe by an interprismatic furrow (2); two lobes and one developed anterior projection separated from the anterior lobe by a well-developed interprismatic furrow (3); three lobes (4).

(46) Distribution of enamel in molars: covering the entire crown (0); interrupted at the base of the lingual wall (1); interrupted at the base and the corner of the lingual wall (2); interrupted at the base and in two strips (3); interrupted along the entire labial wall of the upper molars (lingual of the lower molars) except for the flexus/ids opposite to the hyopflexus/id (4); interrupted along the entire lingual wall and anterolingual and posterolingual walls (5).

(47) Fossettes/ids in late ontogenetic stages: present (0); absent (1).

(48) Fossettes/ids in young-adult ontogenetic stages: present (0); absent (1).

(49) Fossettes/ids in juvenile ontogenetic stages: present (0); absent (1).

(50) Shape of the fossettes/ids: elongated (0); subcircular (1).

(51) Number of lobes in P4: one (0); two (1).

(52) Mesofossettid in young-adult stages: present (0); absent (1).

(53) Fusion of the mesolophid with the anterolophid (metalophulid II and metalophulid I respectively *sensu* Marivaux et al. [6]): no (0); yes (1). (Kramarz [5]: character 15).

(54) Reduction of the mesolophid (metalophulid II sensu Marivaux et al. [6]): complete (0), reduced (1). (Kramarz [5]: character 16).

(Continued)

(55) Mesoflexid remains opened: no (0); yes (1). (Kramarz [5]: character 17).

(56) Size of oval foramen: small (1); large (0). (Quintana [7]: character 1).

(57) Apex of mesopterygoid fossa with respect to M2: level with M2 (0); apex in front of M2 (1). (Quintana [7]: character 2).

(58) Articulation of nasals with respect to premaxillae: nasals articulate with premaxillae throughout their length (0); anterior half of nasals do not articulate with premaxillae (1). (Quintana [7]: character 6).

(59) Shape of frontals: not convex (0); convex (1); markedly convex posteriorly (2). (Quintana [7]: character 15; Ubilla et al. [8]: character 14).

(60) Interorbital width: longer or equal to braincase (0); shorter than braincase (1). (Quintana [7]: character 14).

(61) Extension of pterygoids: short (0); long and oriented posteriorly (1). (Quintana [7]: character 17).

(62) Pterygoids: free (0); fused to the auditory bullae (1). (Quintana [7]: character 18).

(63) Shape of the margins of the incisive foramina: with rounded margins (0); with straight and parallel margins (1). (Quintana [7]: character 21; Ubilla et al. [8]: character 6).

(64) Position of the posterior margin of the upper diastema: not vertical (0); vertical (1). (Quintana [7]: character 23; Ubilla et al. [8]: character 23).

(65) Shape of palate: concave (0); plane (1). (Quintana [7]: character 24; Ubilla et al. [8]: character 12).

(Continued)

(66) Upper zygomatic process of the maxilla: not extended as plate-shaped in the rostrum (0); plate-shaped (1). (Quintana [7]: character 25; Ubilla et al. [8]: character 24).

(67) Shape of upper margin of the infraorbital foramen: not straight (0); with straight upper margin (1). (Quintana [7]: character 26; Ubilla et al. [8]: character 5).

(68) Incisive foramina: not reduced (0); reduced (1). (Quintana [7]: character 27).

(69) Jugular and carotid foramina: fused (0); not fused (1). (Quintana [7]: character 28; Ubilla et al. [8]: character 36).

(70) Position of lacrimal: totally interposed between maxilla and premaxilla (0); partially interposed between maxilla and premaxilla (1). (Quintana [7]: character 32; Ubilla et al. [8]: character 8).

(71) Postfrontal spinous process of the jugal: absent (0); present (1). (Quintana [7]: character 33; Ubilla et al. [8]: character 31).

(72) Orbital foramen: fused to the foramen rotundum (0); separated from the foramen rotundum (1). (Quintana [7]: character 34).

(73) Position of upper incisors: orthodont (0); inclined (1). (Quintana [7]: character 35).

(74) Enamel of upper and lower incisors: uncolored (0); with color (1). (Quintana [7]: character 36; Ubilla et al. [8]: character 13).

(75) Shape of nasals: not anteriorly vaulted (0); greatly vaulted anteriorly (1). (Quintana [7]: character 38; Ubilla et al. [8]: character 3).

(76) Position of parietals: in the same plane with respect to the nasals (0); in different plane with respect to the nasals (1). (Quintana [7]: character 40; Ubilla et al. [8] character 25).

(Continued)

(77) Position of the occipital condyles: below the lower limit of the auditory bullae (0); above the lower limit of the auditory bullae (1). (Quintana [7]: character 41; Ubilla et al. [8]: character 27).

(78) Supraorbital foramen or notch: conspicuous (0); absent (1). (Quintana [7]: character 42).

(79) Masseteric fossa of the zygomatic arch: shallow and not well delimited (0); deep and well delimited (1). (Ubilla et al. [8]: character 9).

(80) Position of the boundary between the mastoid and paraoccipital processes: at the same level or above the external auditory meatus (0); beneath the external auditory meatus (1). (Ubilla et al. [8]: character 15).

(81) Shape of the squamosal process: straight (0); curved (1). (Ubilla et al. [8]: character 18).

(82) Shape of the external auditory meatus: long and tube-shaped (0); short (1). (Ubilla et al. [8]: character 19).

(83) Posterior apophysis of the squamosal: partially covering the epitympanic sinus (0); completely covering the epitympanic sinus (1) [9,10].

(84) Nasolacrimal foramen in the maxilla: not laterally exposed (0); laterally exposed (1) [11].

(85) Length of ulna bone with respect to length of skull: ulna less or same than skull (0); ulna greater than skull (1) [7].

(86) Length of shin bone with respect to length of skull: shin bone less than skull (0); shinbone greater than skull (1) [7].

(Continued)

(87) Length of radius with respect to length of humerus: radius less than humerus (0); radius greater than humerus (1) [7].

(88) Shape of M1-M2 in oclusal view: without secondary external flexus (H.S.E. *sensu* Vucetich et al. [12]), primary external flexus (H.P.E sensu Vucetich et al. [12]) (0); H.S.E. deeper than H.P.E. (1); H.S.E. suequall to H.P.E. and shallow (2); H.P.E. deeper than H.S.E (3); without H.P.E. only H.S.E. (4). (New character)

(89) Depth of accesory fissures in M1-M2: absent (0); secondary external flexus (H.S.E. *sensu* Vucetich et al. [12]) less than half of the lobe (1); H.S.E. reaches half of the lobe (2). (New character)

(90) Number of lobes in M3: 1 (0); 2 (1); 2 + 1 (2); 4 to 5 (3); 6 to 7 (4); more to 7 (5).

(91) m1-m2 of triangular lobe: formed by triangular lobes with wide base (0); formed by triangular lobes with narrow base (1). (New character)

(92) Anterior lobe of m1-m2: heart-shaped (0); laminate (1). (New character)

(93) m1-m2 laminate: complex, with tertiary internal flexid (h.t.i. sensu Vucetich et al., 2005) and secondary external flexid (h.s.e. *sensu* Vucetich et al. [12]) without splitting (0); complex, with flexids crossing the opposit wall (hiploflexiids, fundamental external flexid [h.f.e. sensu Vucetich et al. [12]], and primary internal flexid [h.p.i. sensu Vucetich et al. [12]]). (New character)

(94) Develop of posterior projection of second lobe of M3: absent (0); incipient (1); developed (2); third lobe (3). (New character)

(95) Paraoccipital aphophyses: short (0); long (1). (New character)

(96) Jugal: low (0); high (1). (New character)

**References**

1. Pérez ME, Vucetich MG (2011) A new extinct genus of Cavioidea (Rodentia, Hystricognathi) from the Miocene of Patagonia (Argentina) and the evolution of cavioid mandibular morphology. J Mamm Evol 18:163-183.
2. Pérez ME, Vucetich MG (2012) New remains of *Asteromys punctus* Ameghino (Hystricognathi, Cavioidea) from the late Oligocene of Patagonia (Argentina) and the origin of Cavioidea sensu stricto. Ameghiniana 49:118-125.
3. Woods CA (1972) Comparative myology of jaw, hyoid, and pectoral appendicular regions of new and old world hystricomorph rodents. Bull Am Mus Nat Hist 147:115-198.
4. Wood AE, Patterson B (1959) The rodents of the Deseadan Oligocene of Patagonia and the beginnings of South American rodent evolution. Bull Mus Compar Zool 120:280-428.
5. Kramarz AG (2005)A primitive cephalomyid hystricognath rodent from the early Miocene of northern Patagonia, Argentina. Acta Palaeontol Pol 50:249-258.
6. Marivaux L, Vianey-Liaud M, Jaeger J (2004) High-level phylogeny of early Tertiary rodents: dental evidence. Zool J Linn Soc 142:105-134.
7. Quintana C (1998) Relaciones filogenéticas de roedores Caviinae (Caviomorpha, Caviidae), de América del Sur. Bol R Soc Esp Hist Nat 94:125-134.
8. Ubilla M, Piñeiro G, Quintana C (1999) A new extinct species of the genus Microcavia (Rodentia, Caviidae) from the upper Pleistocene of the northern basin of Uruguay, with paleobiogeographic and paleoenvironmental comments. Stud Neotrop Fauna E 34:141-149.
9. Vucetich MG (1975) La anatomía del oído medio como indicadora de relaciones sistemáticas y filogenéticas en algunos grupos de roedores Caviomorpha. Actas I Congreso Argentino de Paleontología y Bioestratigrafía 447-494.
10. Vucetich MG (1984) Los roedores de la Edad Friasense (Mioceno medio) de Patagonia. Rev Mus La Plata 8:47-126.
11. Ubilla M, Riderknecht A (2003) A late Miocene Dolichotinae (Mammalia, Rodentia, Caviidae) from Uruguay, with comments about the relationships of some related fossil species. J Neotrop Mammal 10:293-302.
12. Vucetich MG, Deschamps CM, Olivares AI, Dozo MT (2005) Capybaras, size, shape, and time: a model kit. Acta Palaeontol Pol 50:259-272.
